# Rab44 isoforms similarly promote lysosomal exocytosis, but exhibit differential localization in mast cells

**DOI:** 10.1002/2211-5463.13133

**Published:** 2021-03-19

**Authors:** Tomoko Kadowaki, Yu Yamaguchi, Kohei Ogawa, Mitsuko Tokuhisa, Kuniaki Okamoto, Takayuki Tsukuba

**Affiliations:** ^1^ Department of Frontier Oral Science Graduate School of Biomedical Sciences Nagasaki University Japan; ^2^ Department of Dental Pharmacology Graduate School of Biomedical Sciences Nagasaki University Japan; ^3^ Department of Dental Pharmacology Graduate School of Medicine Dentistry and Pharmaceutical Sciences Okayama University Japan

**Keywords:** exocytosis, isoforms, large GTPase, mast cells, Rab44

## Abstract

Rab44 is a large Rab GTPase containing a Rab GTPase domain and some additional N‐terminal domains. We recently used Rab44‐deficient mice to demonstrate that Rab44 regulates granule exocytosis in mast cells and IgE‐mediated anaphylaxis. In mouse mast cells, Rab44 is expressed as two isoforms, namely, the long and short forms; however, the characteristics of these two isoforms remain unknown. Here, we investigated secretion and localization of the human long Rab44 isoform and the two mouse isoforms and their mutants expressed in rat basophilic leukemia (RBL)‐2H3 cells. Expression of the human long isoform and both mouse isoforms caused an increase in β‐hexosaminidase secretion. Confocal and quantitative analyses showed that both human and mouse long isoforms localized mainly to lysosomes while the mouse short isoform localized mainly to the ER. Live imaging with LysoTracker indicated that the size and number of LysoTracker‐positive vesicles were altered by the various mutants. Ionomycin treatment partially altered localization of both long isoforms to the plasma membrane and cytosol, whereas it had little effect on colocalization of the short isoform with lysosomes. Mechanistically, both human and mouse Rab44 proteins interacted with vesicle‐associated membrane protein 8 (VAMP8), a v‐SNARE protein. Therefore, Rab44 isoforms similarly promote lysosomal exocytosis, but exhibit differential localization in mast cells.

## Graphical abstract

We investigated the difference in function and localization of the human and mouse Rab44 isoforms and their mutants. Expression of the human and mouse long and short isoforms increased β‐hexosaminidase secretion in rat basophilic leukemia‐2H3 cells. However, both human and mouse long isoforms localized mainly to lysosomes while the mouse short isoform localized mainly to the ER.

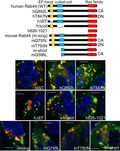

**Abbreviations**
BMMCs: bone marrow‐derived mast cells
CA: constitutively active
DN: dominant negative
hWT: human Rab44
MALDI: matrix‐associated laser desorption/ionization
m‐long: mouse Rab44 long form
m‐short: mouse Rab44 short form
NFATc1: nuclear factor of activated T cells c1
NPY: neuropeptide Y
RBL: rat basophilic leukemia
TNF: tumor necrosis factor
TOF‐MS: time‐of‐flight mass spectrometry
VAMP8: vesicle‐associated membrane protein 8

Rab GTPases are master regulators of intracellular membrane trafficking [1, 2, 3] and are divided into two groups. Members of the first group, consisting of Rab 1–43, are typical “small” Rab GTPases, with molecular weights of approximately 20–30 kDa. The second group consists of Rab44, Rab45 [alias RASEF (RAS and EF‐hand domain‐containing protein)], and CRACR2A (calcium release‐activated channel regulator 2A) [alias Rab46]. The “large” Rab GTPases have been shown to contain many unusual features compared to small Rab GTPases; for example, large Rab GTPases possess additional domains including the EF‐hand, coiled‐coil domains, and Rab GTPase domains [4]. Previously, our research group identified Rab44 as an upregulated protein during osteoclast differentiation [5]. Knockdown of Rab44 enhances osteoclast differentiation, and conversely, overexpression of Rab44 inhibits osteoclast differentiation [5]. In RAW‐D cells, a macrophage cell line, Rab44, is mainly localized in the Golgi complex and lysosomes. Since Rab44 depletion causes an elevation in intracellular Ca^2+^ transients from the lysosomal calcium channel proteins, TRPMLs (transient receptor potential cation channel, mucolipin), it is considered that Rab44 deficiency increases Ca^2+^ influx followed by the activation of nuclear factor of activated T cells c1 (NFATc1), which is a master regulator of osteoclastogenesis, signaling in RANKL‐stimulated macrophages. More recently, by analyzing Rab44‐knockout mice, we reported that Rab44 regulates IgE‐mediated anaphylaxis in mice and granule exocytosis in mast cells [6].

Mast cells are granulocytes that have differentiated from hematopoietic cells in the bone marrow [7]. These are distributed to the respiratory mucosa, gastrointestinal tract, and connective tissues [8]. Mast cells are responsible not only for anaphylaxis, allergy, and asthma, but also for various innate and adaptive immunity, inflammatory, and pathogen defense processes [9]. The main function of mast cells is the release of secretory granules in response to the interaction between IgE and the receptor, FcεRI, on the cell surface [10]. The secretory granules of mast cells are considered to be secretory lysosomes since they contain lysosomal membrane proteins and lysosomal hydrolases such as β‑hexosaminidase and cathepsin D, and biogenic amines such as histamine and serotonin [11]. Upon FcεRI‐mediated activation, the release of secretory granules is controlled by many factors [12]. Membrane translocation, docking, and fusion events with the plasma membrane are controlled by the t‐SNAREs SNAP‐23 and STX4, together with the v‐SNARE vesicle‐associated membrane protein (VAMP) 8, which form stable tetrameric complexes [13, 14]. During these steps, some Rab GTPases act as molecular switches.

To elucidate the physiological function of Rab44, we generated Rab44‐knockout mice, and Rab44‐deficient bone marrow‐derived mast cells (BMMCs) showed reduced FcεRI‐mediated β‐hexosaminidase secretion [6]. In mouse BMMCs, Rab44 was expressed as two isoforms due to alternative splicing [6]. However, the physiological roles of the two isoforms of Rab44 in mast cells remain to be clarified. In this study, we constructed various mutants of human and mouse Rab44 and demonstrated that both isoforms increased FcεRI‐mediated β‐hexosaminidase secretion and that the long forms of human and mouse Rab44 mainly localized to lysosomes, whereas the mouse short form mainly localized to nonendolysosomal compartments. Moreover, we showed that mouse Rab44 interacted with the v‐SNARE protein, VAMP8.

## Methods

### Antibodies and reagents

Mouse monoclonal anti‐GAPDH (M171‐3), anti‐GFP (598), anti‐EEA1 (M176‐3), and anti‐KDEL (M181‐3) antibodies were obtained from Medical & Biological Laboratories (Nagoya, Aichi, Japan). Rat monoclonal anti‐mouse LAMP1 (553792) and mouse monoclonal anti‐GM130 (610823) antibodies were from BD Biosciences (Franklin Lakes, NJ, USA). Rabbit polyclonal anti‐cathepsin D was prepared as described previously [15, 16]. Rabbit monoclonal anti‐VAMP8 antibody (ab76021) was from Abcam (Cambridge, UK). Mouse monoclonal anti‐Rab27B (66944‐1‐Ig), anti‐GFP (66002‐1‐Ig) antibodies were obtained from Proteintech (Rosemont, IL, USA). Alexa Fluor 488‐conjugated goat anti‐rabbit IgG and Alexa Fluor 555‐conjugated goat anti‐mouse, anti‐rat, and anti‐rabbit IgG were from Thermo Fisher Scientific (Waltham, MA, USA). Monoclonal anti‐DNP IgE was from Sigma‐Aldrich. Antibodies against Rab44 were raised in rabbits using a recombinant protein and prepared as previously described [5, 6, 17]. *p*‐Nitrophenyl‐N‐acetyl‐β‐D‐glucosaminide (N9376) and 2,4‐DNP‐human serum albumin (HSA) (A6661) were from Sigma‐Aldrich (Louis, MO, USA). The histamine ELISA kit was from Enzo Life Sciences (NY, USA). Ionomycin and PMA were from FUJIFILM‐WAKO (Tokyo, Japan). The expression vector for mRFP‐tagged NPY was a kind gift from Prof. Mitsunori Fukuda of Tohoku University [18].

Animal experiments conducted in this study were approved by the Nagasaki University Animal Experiment Committee (No. 1703071365). All experiments including recombinant DNA were performed following the guidelines of the Nagasaki University Recombinant DNA Experiment Safety Committee (no. 1607081403).

### Cell culture

The rat basophilic leukemia cells, RBL‐2H3 (JCRB0023), and WEHI‐3 (JCRB9093) cells were provided by the RIKEN BioResource Research Center (Ibaraki, Japan) through the National BioResource Project of the MEXT/AMED, Japan. RBL‐2H3 cells were grown in DMEM containing 10% fetal bovine serum (FBS), 50 U·mL^−1^ penicillin, and 50 μg·mL^−1^ streptomycin, at 37 °C and 5% CO_2_.

Bone marrow cells from 8‐week‐old mice were cultured at 37°C and 5% CO_2_ in RPMI‐1640 medium containing 10% FBS, 2 mm l‐glutamine, 5 × 10^−5^ m 2‐mercaptoethanol, 1% penicillin and streptomycin, and 30% WEHI‐3‐conditioned medium as an IL‐3 source. After 5 d, nonadherent cells were cultured at 1 × 10^5^ cells·mL^−1^ in fresh complete medium containing WEHI‐3‐conditioned medium. After 4 weeks of culture, with the medium exchanged for fresh complete medium every third day, more than 95% of cells were confirmed as BMMCs by toluidine blue staining.

### Quantitative reverse transcription‐PCR

Total RNA was transcribed into cDNA using ReverTra Ace qPCR RT Master Mix (Toyobo). qRT‐PCR was performed using the Brilliant III UltraFast SYBR Green QPCR Master Mix (Agilent Technologies, Santa Clara, CA, USA) and an Applied Biosystems StepOne Plus real‐time PCR system (Thermo Fisher Scientific). Primers for quantitative PCR (supplementary Table S1) were designed using primer3 software (http://primer3.sourceforge.net/). The expression level of each targeted gene was normalized to *GAPDH* expression. All PCRs were performed in triplicate. The efficiency of primer binding was determined using linear regression by plotting the cycle threshold (*C_T_*) value versus the log of the cDNA dilution. Three independent experiments were performed, and these had comparable results.

### Retrovirus construction and expression of Rab44 in RBL‐2H3 cells

The full‐length cDNA of long and short forms of mouse Rab44 were generated by PCR using cDNA derived from bone marrow. The full‐length human Rab44 gene was synthesized and cloned into pcDNA3.1 + N‐DYK by GenScript (Piscataway, NJ, USA). Several mutant *Rab44* genes were produced by PCR using a PrimeSTAR mutagenesis kit (Takara, Shiga, Japan). Primers used for these experiments are listed in Table S1. PCR products were cloned into the pMSCVpuro (Clontech Takara, Shiga, Japan) retroviral vector using an In‐Fusion cloning kit (Clontech Takara). The pMSCVpuro vector with an eGFP fragment inserted, resulting in N‐terminal eGFP‐fusion proteins, was kindly provided by Prof Kosei Ito (Nagasaki University, Japan). Vectors were transfected into HEK293T cells using Lipofectamine 3000 (Life Technologies) The supernatants containing virus particles were collected after 48 h and were used to infect RBL‐2H3 cells. Cells stably expressing Rab44 were selected using puromycin (8 μg·mL^−1^) in the culture medium, with the medium changed every 3 d for 3 weeks.

### Gene silencing by RNA interference

*Rab44* knockdown in BMMCs was performed using Stealth RNAi siRNA. Three 25‐mer Stealth RNAi duplexes targeting mouse Rab44 and a control Stealth RNAi were designed using the RNAi design program (Invitrogen). The sequences were as follows: Stealth RNAi duplex #1: 5′‐CCGAUCCAGCCAAACAGUCCUUAGA‐3′ and 5′‐UCUAAGGACUGUUUGGCUGGAUCGG‐3′; for Stealth RNAi duplex #2: 5′‐CCCGCAUUGUUAGACAGAUCUCCAU‐3′ and 5′‐AUGGAGAUCUGUCUAACAAUGCGGG‐3′ and Stealth RNAi duplex #3: 5′‐GCCAAGUCCUUAGUCUGGAUAGCCU‐3′ and 5’‐AGGCUAUCCAGACUAAGGACUUGGC‐3’. BMMCs were cultured in antibiotic‐free media in a flask for 2 d. The next day, siRNAs were transfected into BMMCs using Lipofectamine RNAiMAX^™^ transfection reagent (Invitrogen, Waltham, MA, USA). The cells were incubated with 50 nm of siRNA for 48 h.

### Degranulation assay

BMMCs and RBL‐2H3 cells (2.5 × 10^5^ cells/well in 48‐well plates) were sensitized for 5 h with 1 µg·mL^−1^ anti‐DNP IgE antibody and were stimulated for 20–60 min with 1 µg·mL^−1^ DNP‐HSA in Tyrode’s buffer (130 mm NaCl, 5 mm KCl, 1.4 mm CaCl_2_, 1 mm MgCl_2_, 5.6 mm glucose, 0.1% BSA, and 10 mm HEPES, pH 7.4). Samples were centrifuged, and supernatants were collected to measure the amounts of β‐hexosaminidase and histamine released. To determine the total intracellular content of β‐hexosaminidase, cells were lysed with 1 % Triton X‐100 in Tyrode’s buffer. For degranulation assays, 50 µL of supernatant or cell lysate was incubated with 200 µL *p*‐nitrophenyl‐*N*‐acetyl‐β‐d‐glucosaminide (1 mm in 0.05 m sodium citrate, pH 4.5) and incubated for 60 min at 37 °C. The reaction was stopped by adding 100 µL of 0.2 m sodium carbonate buffer, pH 10.0, and absorbance was measured at 405 nm using a microplate reader. The percent degranulation was calculated as follows: (absorbance of culture supernatants × 100)/(sum of absorbance of cell lysate and culture supernatant).

### Immunocytochemistry

Cells were cultured on cover glasses and fixed with 4.0% PFA in PBS for 20 min at 25 °C. The aldehyde groups were quenched with ammonium chloride and then washed four times with PBS. The cells were permeabilized with 0.1% Triton X‐100 in PBS for 15 min. Nonspecific binding sites were blocked with 0.2% gelatin in PBS and subsequently incubated with primary antibodies for 1 h at room temperature. The cells were then washed three times with PBS‐gelatin and were incubated with the appropriate secondary antibody. Nuclear staining was then performed using DAPI. The samples were subjected to laser‐scanning confocal microscopy (LSM800, Carl Zeiss, Oberkochen, Germany) and analyzed by Airyscan processing (ZEN2.3 software, Carl Zeiss). Subcellular distribution and colocalization were quantified using Pearson's correlation coefficients. Unpaired *t*‐tests were used to identify differences whenever a significant difference (*P < 0.05, **P < 0.01, or ***P < 0.001) was determined through analysis of variance (ANOVA).

### Live cell imaging

RBL‐2H3 cells expressing the GFP‐Rab44 constructs were grown on a glass‐bottom dish and were fluorescently labeled with 0.1 µm LysoTracker Red DND‐99 (Thermo Fisher) and 5 µg·mL^−1^ Hoechst 33342 (Dojin) at 37 °C for 30 min. The cells were washed with media and observed under a confocal microscope. The area of LysoTracker‐positive vesicles was calculated using ImageJ, and the data were subjected to statistical analysis.

### Immunoprecipitation

Immunoprecipitation was performed according to previously described methods with some modifications [19]. Briefly, cells were washed twice with PBS and then incubated with 2 mm dithiobis(succinimidylpropionate) (DSP) on ice for 2 h. The crosslinking reaction was stopped with 20 mm Tris/HCl, pH 7.5. Cells were then lysed in lysis buffer (50 mm Tris/HCl, pH 7.5, containing 250 mm NaCl, 0.1% Nonidet P‐40, 2 mm EDTA, and proteinase inhibitors). After centrifugation at 25 000 ***g*** for 10 min at 4 °C, the supernatants were collected, and 1 mg (500 µL) of protein was incubated with 2 µL primary antibody for 1 h, followed by overnight incubation with 40 µL of 50% protein G‐Sepharose slurry at 4 °C. The beads were washed four times with lysis buffer and then washed once with PBS. The sedimented beads were resuspended in Laemmli’s sample buffer containing 100 mm DTT and were boiled and subjected to western blot analysis.

### Western blot analysis

Western blot analysis was performed as previously described [20, 21]. Briefly, the cells were lysed in cell lysis buffer. Equal amounts of protein were subjected to SDS/PAGE followed by transfer onto a polyvinylidene difluoride membrane. The blots were blocked with 5% milk in TBS for 1 h at 25 °C, incubated with primary antibodies overnight at 4 °C, washed, incubated with horseradish peroxidase‐conjugated secondary antibodies, and detected using Immobilon Forte Western HRP substrate (Merk‐Millipore, Burlington, MA, USA). Immunoreactive bands were analyzed using a LAS‐4000 Mini imaging system (Fujifilm, Tokyo, Japan).

### Rab44‐knockout mice

Rab44‐knockout mice were generated as previously described [6]. Briefly, we generated mice lacking the *Rab44* gene using CRISPR/Cas9‐mediated genomic editing. *Rab44* ^−/−^ mice (accession no. CDB0039E: http://www2.clst.riken.jp/arg/mutant%20mice%20list.html) developed normally. For various experiments, age‐ and sex‐matched wild‐type mice were used as controls. Mice were housed under specific pathogen‐free conditions in the animal facility of Nagasaki University.

### Statistical analysis

Quantitative data are presented as mean ± standard deviation (SD). Statistical analyses were performed using prism 7 (GraphPad, San Diego, CA, USA).

## Results

### Native Rab44 localizes mainly to lysosomes and the ER and partially to early endosomes in bone marrow mast cells, and Rab44 knockdown impairs FcεRI‐mediated degranulation

**Fig. 1:**
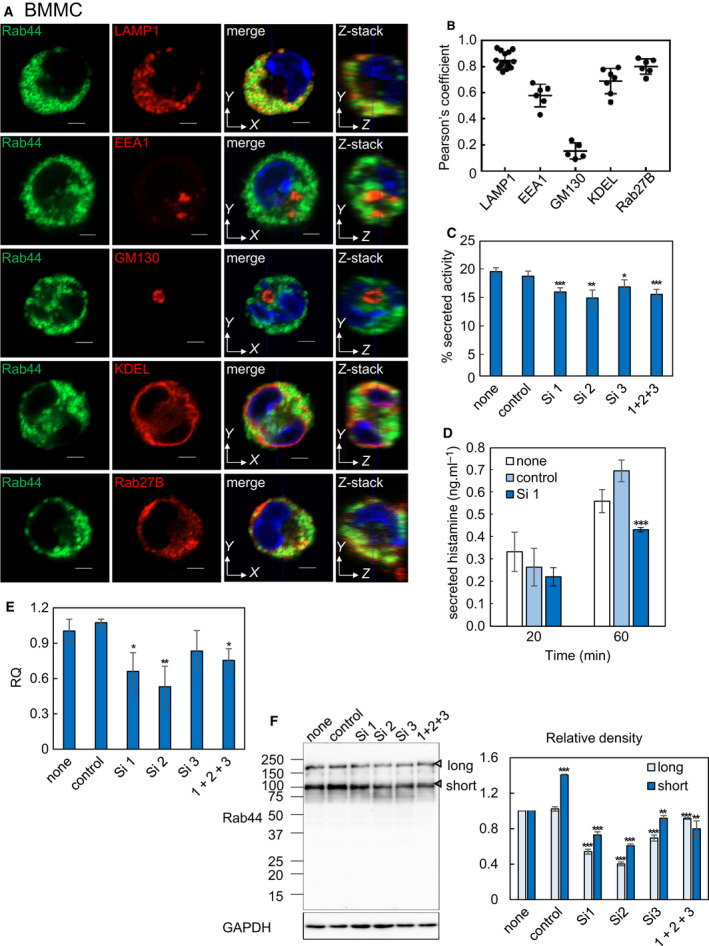
Localization of Rab44 in BMMCs and effects of Rab44 knockdown on FcεRI‐mediated degranulation. (A) Immunofluorescence confocal images of Rab44, LAMP1 (a marker for late endosomes/lysosomes), EEA1 (a marker for early endosomes), GM130 (a marker for the Golgi complex), KDEL (a marker for ER), and Rab27B (a small Rab protein regulating exocytosis in mast cells) in BMMCs. YZ image was reconstructed at the blue line in XY image. Bar: 2 μm. (B) Quantitative analysis of the ratio of colocalization of Rab44 with organelle markers and Rab27B. Data are mean ± SD (*n* ≥ 5). (C) Secreted β‐hexosaminidase levels in siRNA‐transfected BMMCs sensitized with mouse monoclonal anti‐DNP IgE and then challenged with DNP. (D) Secreted histamine levels determined by ELISA in siRNA‐transfected BMMCs sensitized with mouse monoclonal anti‐DNP IgE and then challenged with DNP. (E) Knockdown efficacy of *Rab44*‐specific siRNA was evaluated by measuring *Rab44* mRNA levels. (F) Western blot analysis of the expression levels in siRNA‐transfected BMMCs using anti‐Rab44 antibody. Relative densitometric analysis normalized by GAPDH of long and short forms in siRNA‐transfected BMMCs compared with nontransfected BMMCs. (C–F) Data are presented as mean ± SD (*n* ≥ 6) and statistically analyzed by Tukey’s multiple comparison test. *P < 0.05, **P < 0.01, ***P < 0.001.

To determine the subcellular localization of endogenous Rab44 in native BMMCs, we analyzed the localization of Rab44 compared with several organelle marker proteins. Rab44 predominantly colocalized with LAMP1 (late endosomes/lysosomes) and KDEL (endoplasmic reticulum; ER) and partially with EEA1 (early endosomes), but not with GM130 (Golgi) (Fig. 1A). In addition, we determined colocalization of Rab44 and Rab27B, which also regulates exocytosis of native mast cells (Fig. 1A). Z‐section analysis of these colocalization patterns also was performed (Fig. 1A). Quantitative analysis of colocalization between Rab44 and these organelle markers is shown in Fig. 1B. These results indicate that native Rab44 localizes mainly to lysosomes and the ER and partially to early endosomes in BMMCs, and Rab44 also considerably colocalizes with Rab27B.

To evaluate Rab44 effects on degranulation, we performed knockdown experiments using small interfering RNA (siRNA) transfection of native BMMCs. Following FcεRI‐mediated stimulation, β‐hexosaminidase secretion significantly decreased in Rab44‐knockdown BMMCs compared to BMMCs transfected with a control siRNA (Fig. 1C). siRNA‐mediated knockdown of Rab44 also decreased histamine secretion by BMMCs by approximately 60% compared to control siRNA‐transfected BMMCs (Fig. 1D). Knockdown efficacy in BMMCs was confirmed by reverse transcription (RT)‐PCR (Fig. 1E). Compared with the control siRNA, #1, #2, #3 and combinations of siRNA reduced Rab44 mRNA expression levels by approximately 40, 50, 15, and 20%, respectively (Fig. 1E). Similar results were observed in the protein expression confirmed by western blotting and densitometric analysis of the same siRNA experiments (Fig. 1F and Fig. S3). Thus, Rab44 knockdown in BMMCs impairs FcεRI‐mediated degranulation, including the secretion of β‐hexosaminidase and histamine. These findings are consistent with our recent results that Rab44‐deficient BMMCs reduce IgE‐mediated degranulation [6].

### Human Rab44 and its mouse two isoforms increase FcεRI‐mediated β‐hexosaminidase secretion in RBL‐2H3 cells

**Fig. 2:**
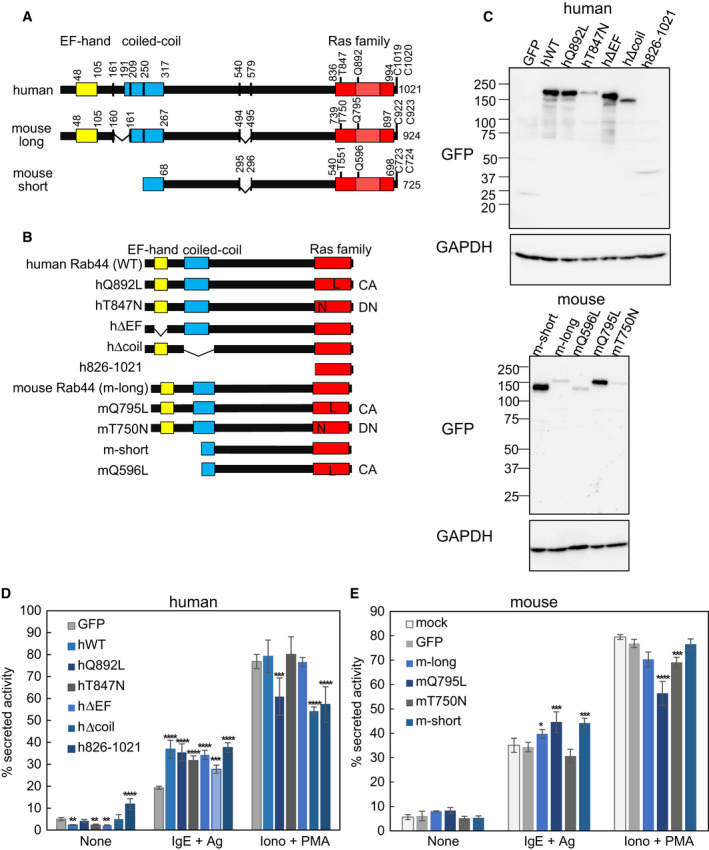
Effects of exogenous expression of wild‐type and mutant Rab44 on FcεRI‐mediated β‐hexosaminidase secretion in RBL‐2H3 cells. (A) Transcripts of wild‐type *human* and *mouse Rab44*. Rab44 was expressed as two isoforms by alternative splicing in mouse BMMCs. The EF‐hand, coiled‐coil, and Ras family domains are shown in yellow, blue, and red, respectively. (B) Schematic representation of wild‐type and mutant Rab44 expressed in RBL‐2H3 cells. The recombinant Rab44 was expressed as GFP‐fusion proteins on their N‐terminus. (C) Rab44 protein expression in RBL‐2H3 cells was confirmed by western blotting analysis using an anti‐GFP antibody. (D–E) FcεRI‐mediated and ionomycin/PMA‐stimulated β‐hexosaminidase secretion assay using RBL‐2H3 cells expressing human (D) and (E) mouse Rab44. Data are presented as mean ± SD (*n* ≥ 6). Asterisks indicate statistical significance compared to GFP. *P < 0.05, ***P < 0.001, and ****P < 0.0001 by Tukey’s multiple comparison test.

Our recent studies indicated that mouse Rab44 is expressed as two isoforms: the long form with a molecular mass of about 160 kDa and the short form of about 95 kDa [6, 17]. Figure 2A shows the molecular structure of human Rab44 and the two forms of mouse Rab44. The cDNA sequences of mouse Rab44 were confirmed by analyzing the sequences from bone marrow cells and BMMCs.

To examine differences among human Rab44 and its mouse two isoforms, we expressed various Rab44 mutants in the rat basophilic leukemia cell line, RBL‐2H3, which is the most useful model for studying mast cells [22, 23, 24]. We constructed wild‐type human Rab44 (hWT) and its mutants, such as a constitutively active (CA) mutation of the Rab domain (hQ892L), a dominant negative (DN) mutation of the Rab domain (hT847N), an EF‐hand motif deletion (hΔEF), a coiled‐coil domain deletion (Δcoil), and a Rab domain only mutation (h826‐1021) as GFP‐fusion proteins through site‐directed mutagenesis (Fig. 2B). Moreover, we constructed mouse wild‐types and various mutants such as mouse Rab44 long form (m‐long), constitutively active (CA) long form (mQ795L), dominant negative (DN) long form (mT750N), short form (m‐short), and CA short form (mQ596L) mutants (Fig. 2B). In RBL‐2H3 cells, endogenous rat Rab44 was detectable as a long form by western blotting and RT‐PCR (Fig. S1).

Although the protein levels of the mutants expressed in RBL‐2H3 cells were different, the expressed proteins were detected based on their molecular weights (Fig. 2C and Fig. S4). Owing to the different expression levels of these constructs, we evaluated their effects on secretion individually by comparing with the GFP control.

Next, we performed an FcεRI‐mediated and ionomycin/PMA‐stimulated β‐hexosaminidase secretion assay using RBL‐2H3 cells expressing the different constructs. In human Rab44‐expressing cells, upon FcεRI‐mediated stimulation, ectopic expression of hWT, hΔEF, hΔcoil, h826‐1021, hQ892L, and hT847N Rab44 proteins caused enhanced β‐hexosaminidase secretion compared with the GFP control (Fig. 2D). It should be noted that the h826‐1021 mutant, containing the Rab domain only, enhanced β‐hexosaminidase secretion under nonstimulated conditions, suggesting that non‐Rab domains have regulatory activity on FcεRI‐mediated β‐hexosaminidase secretion (Fig. 2D). Upon ionomycin/PMA stimulation, the expression of hWT, hΔEF, and hT847N proteins induced comparable β‐hexosaminidase secretion to that of the GFP control (Fig. 2D). However, the expression of hQ892L, hΔcoil, and h826‐1021 mutants resulted in lower secretion levels compared with that of the GFP control (Fig. 2D).

In mouse Rab44‐expressing cells, ectopic expression of the m‐short and mQ795L mutants significantly enhanced FcεRI‐mediated β‐hexosaminidase secretion compared with the GFP control (Fig. 2E). Similarly, expression of the m‐long mutant significantly increased β‐hexosaminidase secretion (Fig. 2E). However, expression of mT750N mutant had little or slightly inhibitory effects on β‐hexosaminidase secretion (Fig. 2E). Upon ionomycin/PMA stimulation, expression of the m‐long and mT750N mutants slightly decreased β‐hexosaminidase secretion compared with the GFP control (Fig. 2E). Moreover, expression of the mQ795L mutant decreased β‐hexosaminidase secretion by ionomycin/PMA stimulation (Fig. 2E). However, expression of the m‐long and m‐short mutant led to a similar secretion level to that of the GFP control (Fig. 2E) by ionomycin/PMA. These results indicate that the long and short forms of Rab44 promote FcεRI‐mediated β‐hexosaminidase secretion.

**Fig. 3:**
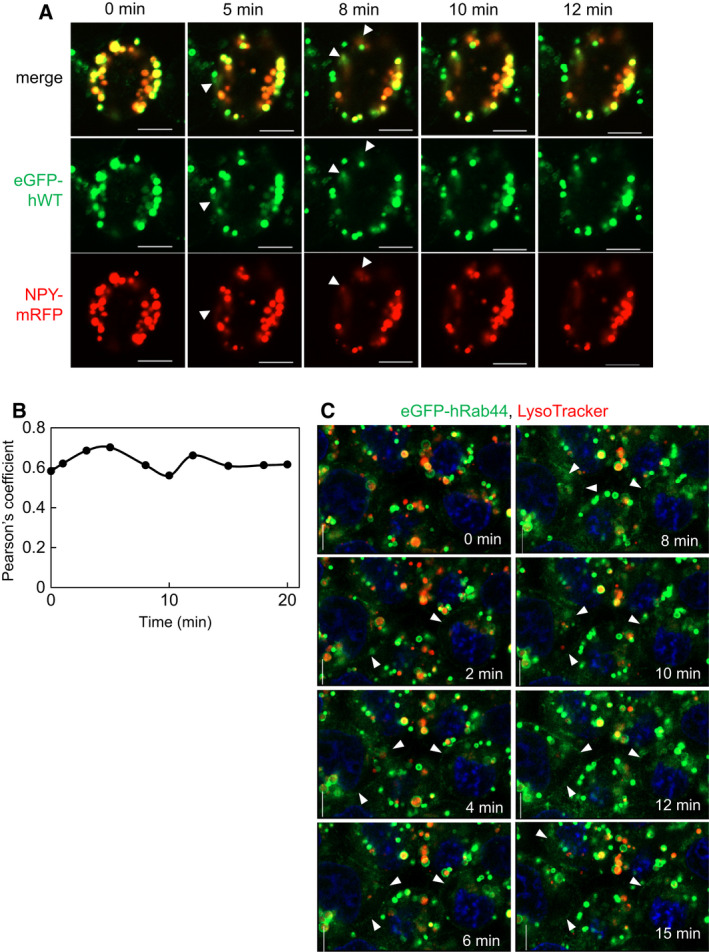
Time‐lapse experiments of FcεRI‐mediated secretion in RBL‐2H3 cells. (A) Time‐lapse confocal microscopic imaging of NPY‐mRFP secretion from RBL‐2H3 cells stably expressing wild‐type eGFP‐human Rab44 (hWT). The cells were sensitized with anti‐DNP IgE and then challenged with DNP‐HSA for the indicated periods. White arrowheads indicate the regions Rab44 and NPY have diffused. Bar: 5 μm. (B) Quantitative analysis of the ratio of colocalization of hWT with NPY‐mRFP. (C) Time‐lapse confocal microscopic imaging of LysoTracker‐stained RBL‐2H3 cells stably expressing wild‐type eGFP‐human Rab44 (hWT). White arrowheads indicate the regions where Rab44 has accumulated in a streak. Bar: 5 μm.

Next, we performed time‐lapse experiments of FcεRI‐mediated secretion using RBL‐2H3 cells expressing eGFP‐hWT and neuropeptide Y (NPY) fused with mRFP (Fig. 3A). Initially, we observed yellow dots, which indicated an overlap between the green fluorescence signal of Rab44 and the red fluorescence signal of NPY. During the 5‐ to 12‐min time course, the red signal gradually became diffuse as indicated by arrowheads, indicating NPY secretion. At the site where the red NPY was diffused, the green hWT was also diffused in synchronization (Fig. 3A, arrowheads). Quantitative analysis of the colocalization of hWT and NPY is shown in Fig. 3B. Stimulation with IgE and antigen caused increased colocalization during the 0–5 min time course, subsequently decreased during the 5–10 min time course, after which it changes and then remains constant (Fig. 3B).

We also performed time‐lapse experiments of hRab44 WT fused with eGFP during FcεRI‐mediated secretion in LysoTracker‐stained RBL‐2H3 cells (Fig. 3C). Before antigen stimulation, hWT (green) and LysoTracker (red) did not overlap; however, hWT initially surrounds the LysoTracker‐positive vesicles (time 0). Immediately after stimulation, the green hRab44 moved sparsely to the cytosol, with streaks appearing at 2 min as indicated by the arrowheads. hRab44 then accumulated into the streaks that seemed to be the plasma membrane, as indicated by the arrowheads, and LysoTracker‐positive acidic vesicles decreased at 4–12 min of the time course. At 15 min, the green linear structure disappeared, and LysoTracker‐stained red vesicles have reappeared.

### Long forms of Rab44 localize mainly to lysosomes, whereas the mouse short form localizes mainly to the ER

**Fig. 4:**
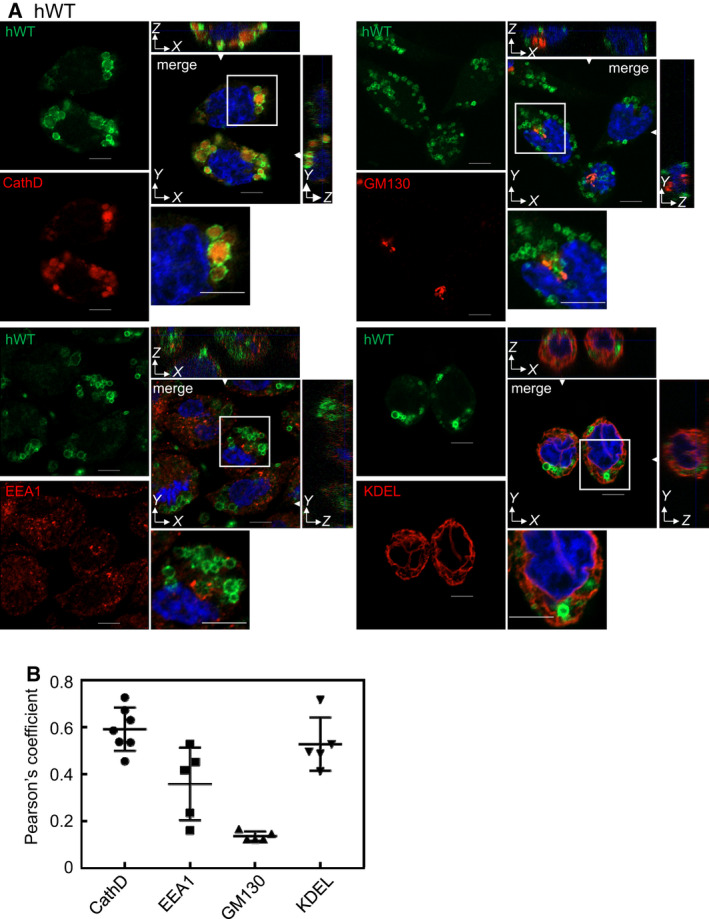
Colocalization of human Rab44 with the organelle markers in RBL‐2H3 cells. (A) Confocal laser microscopic analysis of RBL‐2H3 cells expressing eGFP‐hWT. XZ and YZ images reconstructed at the line indicated with white arrowheads in XY image were shown. Cells were immunofluorescently stained for cathepsin D (marker for late endosomes/lysosomes), EEA1 (marker for early endosomes), GM130 (marker for the Golgi), and KDEL (marker for ER). Bar: 5 μm. (B) Quantitative analysis of the colocalization ratio of hWT with these organelle markers. Data are mean ± SD (*n* ≥ 5).

We investigated the localization of human and mouse Rab44 and their mutants in RBL‐2H3 cells. hWT formed donut‐like structures that mainly colocalized with cathepsin D (Fig. 4A). The hWT also colocalized with KDEL, and partially with EEA1, but not with GM130 (Fig. 4A). Z‐stack analysis was also performed (Fig. 4A). Quantitative analysis showed that the colocalization score of the hWT with cathepsin D was the highest, those with KDEL and EEA1 were moderate, and those with GM130 were quite low (Fig. 4B).

**Fig. 5:**
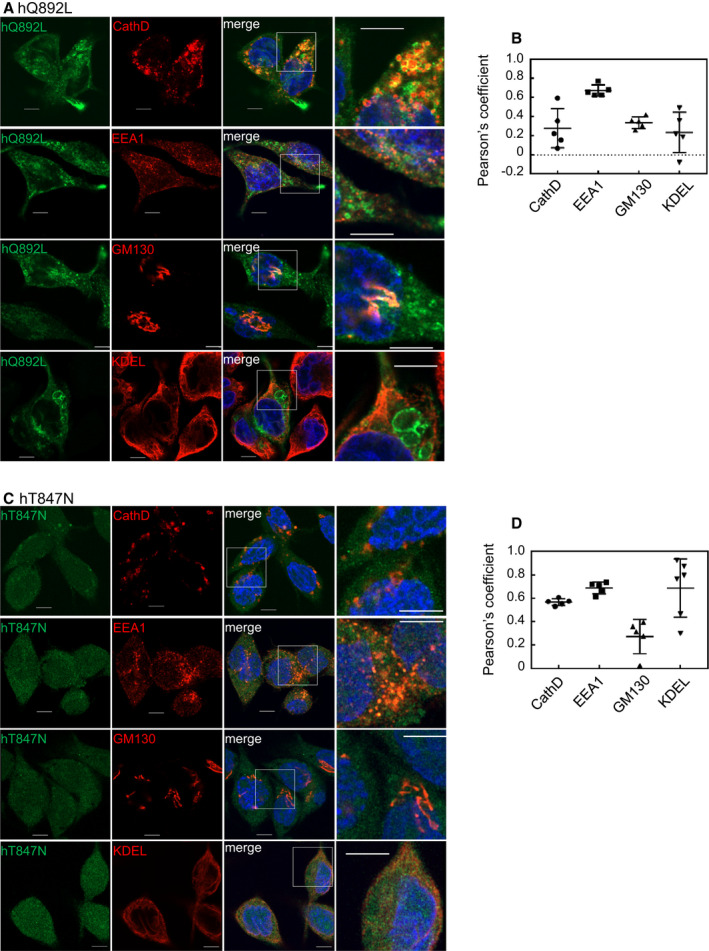
Subcellular localization of CA and DN mutants of human Rab44 in RBL‐2H3 cells. (A,B) eGFP‐hQ892L (CA), (C,D) eGFP‐hT847N (DN). (A, C): Confocal laser microscopic analysis of RBL‐2H3 cells immunofluorescently stained for cathepsin D, EEA1, GM130, and KDEL. Bar: 5 μm. (B, D): Quantitative analysis of the colocalization ratio of these mutants with these organelle markers. Data are mean ± SD (*n* ≥ 5).

The CA mutant, hQ892L, was exclusively observed in small vesicles and was detectable at the cell edges (Fig. 5A). hQ892L‐labeled vesicles were mainly merged with EEA1 (Fig. 5A). As a characteristic observation, hQ892L accumulated in the protrusions of the cells (Fig. 5A). Quantitative analysis indicated that the colocalization score of the hQ892L mutant with EEA1 was higher and those with other organelle markers were lower (Fig. 5B).

The DN mutant hT847N was mostly diffuse in the cytosol, and it partially colocalized with cathepsin D, EEA1, and KDEL, but not with the GM130 (Fig. 5C). We further confirmed the hT847N localization together with cathepsin D using anti‐GFP antibody to enhance the hT847N expression (Supplementary Figure 2A). Immunocytochemistry with anti‐GFP antibody indicated diffused pattern in the cytosol and the partial colocalization with cathepsin D, as same as the detection of fluorescence of GFP (Fig. S2A). Quantification of colocalization between the hT847N mutant and these organelle markers validated these observations (Fig. 5D).

**Fig. 6:**
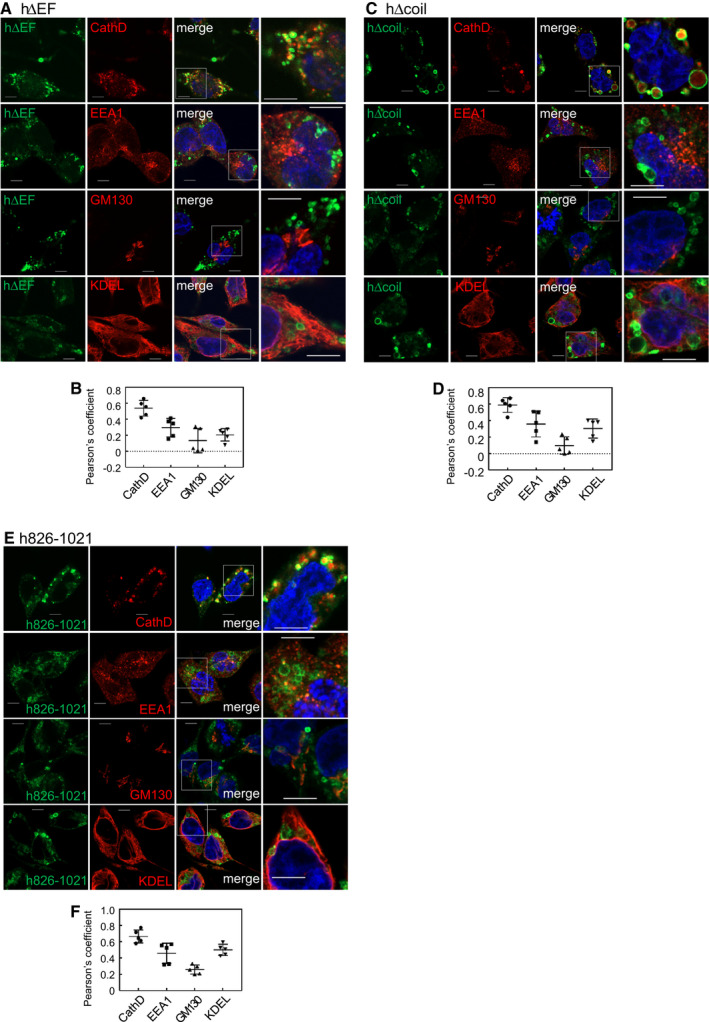
Colocalization of the deletion mutants of human Rab44 with the organelle markers in RBL‐2H3 cells. (A,B) eGFP‐hΔEF, (C,D) eGFP‐hΔcoil, (E,F) eGFP‐h826‐1021. (A, C, E): Confocal laser microscopic analysis of RBL‐2H3 cells immunofluorescently stained for cathepsin D, EEA1, GM130, and KDEL. Bar: 5 μm. (B, D, F): Quantitative analysis of the colocalization ratio of these mutants with these organelle markers. Data are mean ± SD (*n* ≥ 5).

The hΔEF mutant formed donut‐like structures that mainly colocalized with cathepsin D (Fig. 6A). Quantification of colocalization of the hΔEF mutant with these organelle markers also showed higher colocalization scores with cathepsin D and lower scores with other organelle markers (Fig. 6B).

The hΔcoil mutant surrounded cathepsin D and caused larger structures (Fig. 6C). Quantitative analysis showed that the colocalization score of the hΔcoil mutant with cathepsin D was the highest, and those with EEA1 and KDEL were moderate, and that with GM130 was quite low (Fig. 6D).

h826‐1021 mutant colocalized mainly with cathepsin D, partially with EEA1 and KDEL, and hardly with GM130 (Fig. 6E). Quantification of the colocalization of the h826‐1021 mutant with these organelle markers was consistent with these observations (Fig. 6F).

**Fig. 7:**
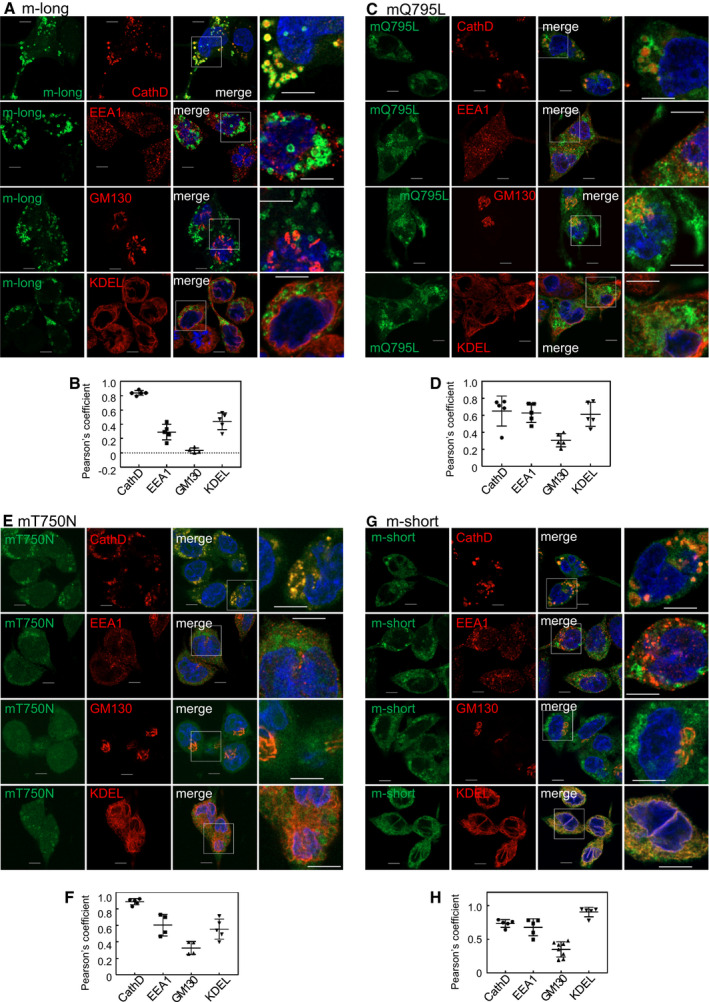
Colocalization of mouse Rab44 and its mutants with the organelle markers in RBL‐2H3 cells. (A,B) eGFP‐m‐long, (C,D) eGFP‐mQ795L, (E,F) eGFP‐mT750N, and (G,H) eGFP‐m‐short. (A, C, E, G): Confocal laser microscopic analysis of RBL‐2H3 cells immunofluorescently stained for cathepsin D, EEA1, GM130, and KDEL. Bar: 5 μm. (B, D, F, H): Quantitative analysis of the colocalization ratio with these organelle markers. Data are mean ± SD (*n* ≥ 5).

The m‐long Rab44 protein formed donut‐like structures that mainly colocalized with cathepsin D was weakly detected in KDEL and EEA1 and failed to colocalize with GM130 (Fig. 7A). Quantitative analysis showed that the colocalization score of the m‐long with cathepsin D was the highest among these markers, those with EEA1 and KDEL were lower, and that with GM130 was almost zero (Fig. 7B). Distribution patterns of m‐long were similar to those of hWT, although the colocalization ratio of m‐long with cathepsin D was higher than that of hWT.

The CA mutant, mQ795L, colocalized partially with cathepsin D, EEA, and KDEL, and rarely with GM130 (Fig. 7C). The mQ795L mutant was weakly detected at the plasma membrane and cell edge, similar to the pattern observed in hQ892L. Quantitative analysis showed that the colocalization scores of mQ795L with cathepsin D, EEA1, and KDEL were almost the same, whereas its colocalization score with GM130 was very low (Fig. 7D). Colocalization of mQ795L with EEA1 was higher than that of m‐long (Fig. 7D).

The DN mutant, mT750N, was mostly diffused, similar to hT847N, and partially formed granular patterns that merged with cathepsin D (Fig. 7E). We further confirmed the mT750N localization together with cathepsin D using anti‐GFP antibody to enhance the hT750N expression (Fig. S2B). Also, immunocytochemistry with anti‐GFP antibody indicated diffused pattern in the cytosol and the partial colocalization with cathepsin D, as same as the detection of fluorescence of GFP (Fig. S2B). Quantitative analysis showed that the colocalization score of mT750N with cathepsin D was highest, those with EEA1 and KDEL intermediate, and that with GM130 was lower (Fig. 7F).

The m‐short form colocalized highly with KDEL and partially with cathepsin D and EEA1 (Fig. 7G). Quantitative analysis displayed that the colocalization score of m‐short and KDEL was higher, those with cathepsin D and EEA1 were intermediate and that with GM130 was lower (Fig. 7H). The distribution profile was similar to that of mQ795L.

The CA mutant, mQ596L, was mainly detected in cathepsin D, but was not observed at the cell edge where the mQ795L mutant localized (Data not shown).

### Size and number of LysoTracker‐labeled vesicles are altered by the expression of Rab44

**Fig. 8:**
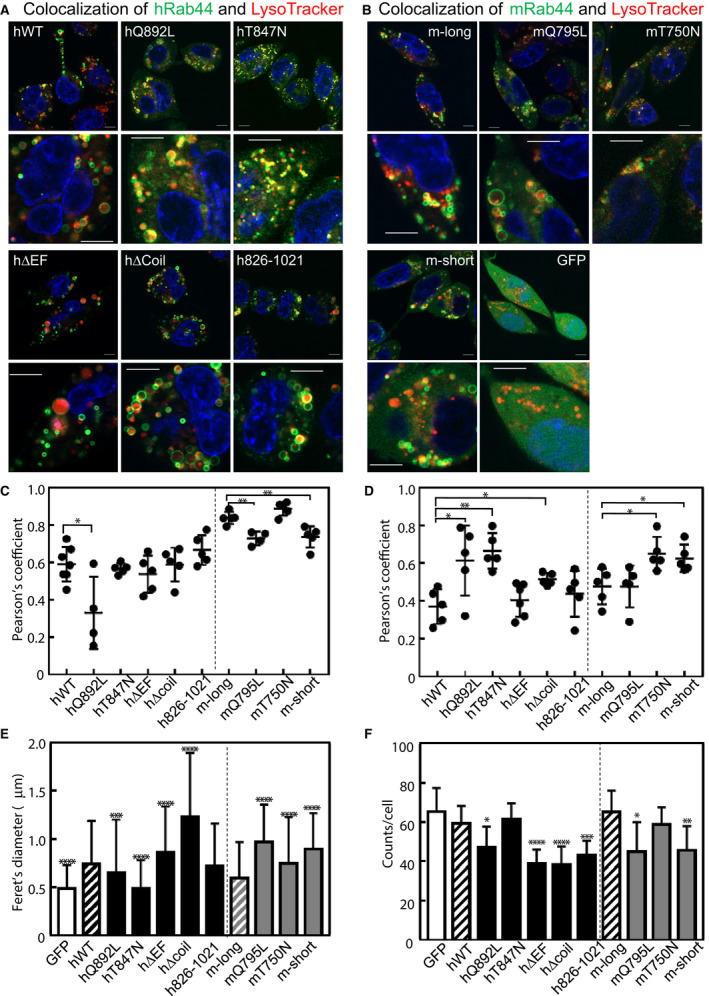
Colocalization of human or mouse Rab44 and its mutants with LysoTracker in RBL‐2H3 cells. (A,B) Confocal laser microscopic analysis of live RBL‐2H3 cells stained with LysoTracker Red. Bar: 5 μm. (A) human Rab44 and its mutants; (B) mouse Rab44 and its mutants. (C) Summary of quantitative analysis of colocalization with cathepsin D (shown in Figs 4, 5, 6, 7). (D) Quantitative analysis of colocalization with LysoTracker (shown in Fig. 8A, B). Asterisks indicate statistical significance compared to hWT or m‐long *P < 0.05, **P < 0.01. (E) Size of LysoTracker‐positive vesicles in the live RBL‐2H3 cells. Data are mean ± SD (*n* ≥ 500). The asterisks indicate statistical significance compared to hWT or m‐long, *P < 0.05, ***P < 0.001, and ****P < 0.0001. (F) Number of LysoTracker‐positive vesicles in the live RBL‐2H3 cells. Data are presented as mean ± SD (*n* ≥ 12). The asterisks indicate statistical significance compared to the hWT or the m‐long, *P < 0.05, **P < 0.01. ***P < 0.001 and ****P < 0.0001. Statistical analyses were performed by unpaired *t*‐tests using graphpad prism 7.

Next, we analyzed the formation of acidic compartments in live RBL‐2H3 cells expressing human and mouse Rab44 and their mutants. Live imaging of these constructs in living cells using LysoTracker showed that the sizes of LysoTracker‐positive vesicles were different for each construct in cells (Fig. 8A, B). The results of observations in live cells (Fig. 8) were similar to those in fixed cells. However, the patterns of colocalization of these Rab44 constructs with cathepsin D and LysoTracker were different since mast cells contain heterogeneous vesicles such as serotonin, cathepsin D‐positive lysosomes, and histamine and tumor necrosis factor (TNF)‐positive granules [7]. Therefore, LysoTracker probably almost detects these acidic compartments.

We compared the colocalization of various Rab44 constructs with cathepsin D‐ or LysoTracker‐positive compartments. Quantification of colocalization with cathepsin D‐labeled lysosomes showed that the colocalization score of hQ892L was significantly lower than that of hWT, while those of other mutants such as hT847N, hΔEF, hΔcoil, and h826‐1021 were similar to that of hWT (Fig. 8C). In mouse Rab44‐expressing cells, the colocalization scores of mQ795L and m‐short were significantly lower than that of m‐long, whereas that of mT750N was similar to that of m‐long (Fig. 8C).

We also investigated colocalization with LysoTracker‐positive compartments. Quantitative analysis revealed that the colocalization scores of hQ892L, hT847N, and hΔcoil were significantly higher than that of hWT, while those of other mutants including hΔEF and h826‐1021 were similar to that of hWT (Fig. 8D). In mouse Rab44 expressing cells, the colocalization scores of mT750N and m‐short were significantly lower than that of m‐long, whereas that of mQ795L was similar to that of m‐long (Fig. 8D).

Quantitative analysis of the sizes of LysoTracker‐labeled vesicles in live cells displayed that hWT formed significantly larger LysoTracker‐labeled vesicles compared with the GFP‐only control (Fig. 8E). Both the CA mutant hQ892L and the DN mutant hT847N formed smaller LysoTracker‐positive vesicles than hWT, whereas hΔEF and Δcoil induced larger LysoTracker‐positive vesicles than hWT (Fig. 8E). However, the h826‐1021 mutant formed vesicles similar in size to that of hWT (Fig. 8E). In mouse Rab44‐expressing cells, mQ795L, mT750N, and m‐short all induced larger LysoTracker‐labeled vesicles than m‐long (Fig. 8E).

When we quantified the number of LysoTracker‐positive vesicles in live cells, the number of the vesicles in cells expressing hWT or the hT847N mutant was almost the same as that in cells expressing GFP alone (Fig. 8F). However, the numbers of vesicles in cells expressing the hQ892L, hΔEF, Δcoil, and h826‐1021 mutants were significantly lower than that of hWT (Fig. 8F). Consistent with the results from human Rab44, the numbers of LysoTracker‐labeled vesicles in cells expressing mQ795L and m‐short were significantly lower than that of m‐long (Fig. 8F). However, the number of vesicles in cells expressing mT750N was similar to that of m‐long (Fig. 8F).

### Ionomycin treatment partially alters localization of both long forms, while it hardy alters the localization of the short form

To explore whether Ca^2+^ influx changes localization of Rab44 and its mutants, we examined the effects of ionomycin on Rab44 localization in cathepsin D‐positive lysosomes.

**Fig. 9:**
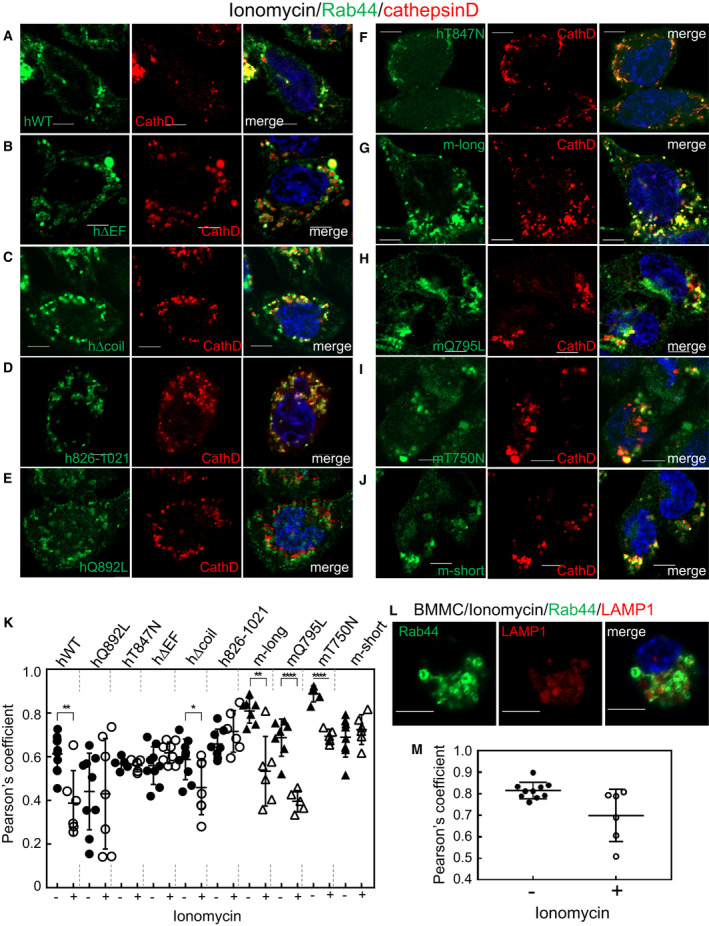
Ca^2+^ influx induced by ionomycin altered Rab44 localization. (A–J) Confocal laser microscopic analysis of eGFP‐Rab44‐expressing RBL‐2H3 cells incubated with ionomycin and immunostained with anti‐cathepsin D antibody. Bar: 5 μm. (K) Quantitative analysis of the colocalization ratio of Rab44 with cathepsin D. (L) Confocal laser microscopic analysis of BMMCs incubated with ionomycin and immunofluorescently stained with anti‐Rab44 (green) and anti‐LAMP1 (red) antibodies. Bar: 5 μm. (M) Quantitative analysis of the ratio of colocalization of Rab44 with LAMP1 in BMMCs. *P < 0.05.

Ionomycin treatment partially altered the localization of hWT to the plasma membrane and the cytosol, although hWT was mainly detected in the lysosomes in nonstimulated cells (Fig. 9A). Quantitative analysis of colocalization of hWT with cathepsin D‐positive lysosomes revealed that ionomycin treatment significantly decreased the colocalization score of hWT with cathepsin D‐positive lysosomes (Fig. 9K).

Even after ionomycin treatment, the hΔEF mutant notably remained in donut‐like structures, which were similar to its localization without stimulation (Fig. 9B). Consistent with this observation, quantification of colocalization of hΔEF with cathepsin D‐labeled lysosomes showed that ionomycin treatment failed to change the colocalization score of hΔEF with cathepsin D‐positive without stimulation (Fig. 9K).

Upon ionomycin treatment, the hΔcoil mutant localized in nonlysosomal vesicles, although the hΔcoil mutant formed larger lysosomal‐like structures without stimulation (Fig. 9C). Quantitative analysis of colocalization of hΔcoil with cathepsin D‐positive lysosomes showed that ionomycin treatment significantly reduced the colocalization score of hΔcoil with cathepsin D‐positive lysosomes (Fig. 9K).

Ionomycin treatment caused little changes in the localization of the mutants, such as h826‐1021, hQ892L, and hT847N (Fig. 9D, E, and F). These findings were confirmed by quantitative analysis of colocalization of these mutants with cathepsin D‐positive lysosomes (Fig. 9K).

In m‐long‐expressing cells, ionomycin treatment caused a partially diffused pattern of m‐long localization, most likely in the cytosol, in spite of the partial colocalization of m‐long with cathepsin D‐positive lysosomes (Fig. 9G). Quantification of colocalization of the m‐long with cathepsin D‐labeled lysosomes showed that ionomycin treatment significantly decreased the colocalization score of m‐long with cathepsin D‐positive lysosomes (Fig. 9K).

Upon ionomycin treatment, the CA mutant, mQ795L, was translocated into nonlysosomal vesicles (Fig. 9H). Quantitative analysis of colocalization of mQ795L with cathepsin D‐labeled lysosomes indicated that ionomycin treatment significantly decreased the colocalization score of the mQ795L with cathepsin D‐positive lysosomes (Fig. 9K).

Ionomycin treatment caused partial localization of the DN mutant mT750N in nonlysosomal vesicles (Fig. 9I). Quantification of colocalization of mT750N with cathepsin D‐labeled lysosomes showed that ionomycin treatment significantly decreased the colocalization score of mT750N with cathepsin D‐positive lysosomes (Fig. 9K).

Importantly, ionomycin treatment hardly altered colocalization of m‐short Rab44 with cathepsin D‐positive lysosomes (Fig. 9J). Quantitative analysis indicated that there were no differences in the colocalization scores of the m‐short with cathepsin D‐positive lysosomes when compared to those with and without ionomycin treatment (Fig. 9K).

We further investigated whether ionomycin treatment alters colocalization of native Rab44 with LAMP1‐positive lysosomes in BMMCs (Fig. 9L). Ionomycin treatment caused partial localization of the native Rab44 in nonlysosomal vesicles (Fig. 9L). Quantification of colocalization showed that ionomycin treatment significantly decreased the colocalization score of Rab44 with LAMP1‐positive lysosomes (Fig. 9M).

Thus, upon ionomycin treatment, Rab44 is likely to have altered its localization, and the EF‐hand domain of Rab44 is likely to be important for Ca^2+^‐mediated translocation.

### Rab44 interacts with the v‐SNARE protein, VAMP8

**Fig. 10:**
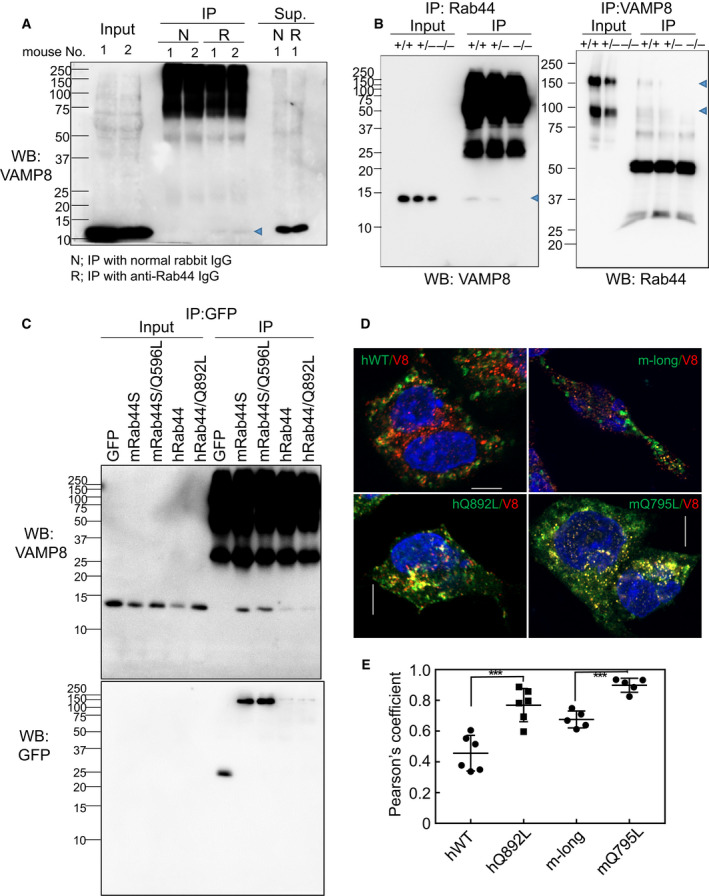
Interaction of Rab44 with VAMP8. (A) BMMC cell lysates from two independent mice (No. 1 and No. 2) were subjected to crosslinking by DSP and immunoprecipitation using anti‐Rab44 IgG (R) or normal rabbit IgG (N). The immunoprecipitate (IP) and nonadsorbed supernatants (Sup) were analyzed using western blotting using an anti‐VAMP8 antibody. The 1/20 amount was loaded on the lanes for both IP and Sup. Blue arrowhead indicates the bands corresponding to VAMP8. (B) BMMC cell lysates of *Rab44^+/+^*, *Rab44^+/−^*, and *Rab44^−/−^* mice were subjected to co‐immunoprecipitation followed by western blotting. (Left panel) IP, anti‐Rab44 IgG; WB, anti‐VAMP8 antibody. (Right panel) IP, anti‐VAMP8 antibody; WB, anti‐Rab44 IgG. Blue arrowheads indicate the bands of VAMP8 (left panel) and Rab44 (right panel). (C) Cell lysates of RBL‐2H3 cells expressing mouse short, mQ596L, human WT, and hQ892L Rab44 were immunoprecipitated with an anti‐GFP antibody and subjected to western blotting using an anti‐VAMP8 antibody. (D) Colocalization of Rab44 with VAMP8. Confocal microscopic analysis of RBL‐2H3 cells expressing eGFP‐hWT, hQ892L, m‐long and mQ795L immunofluorescently stained with an anti‐VAMP8 antibody (red). Bar: 5 μm. (E) Quantitative analysis of the ratio of colocalization of human and mouse Rab44 wild‐type or their CA mutants with VAMP8‐positive compartments. Data are mean ± SD (*n* ≥ 5). ***P < 0.001. Statistical analyses were performed by unpaired *t*‐tests using graphpad prism 7.

To examine the molecular mechanisms involved in the regulation of FcεRI‐mediated β‐hexosaminidase secretion by Rab44, we investigated the proteins interacting with Rab44. We first screened the molecules that interact with Rab44 in BMMCs by immunoprecipitation using an anti‐Rab44 antibody followed by matrix‐associated laser desorption/ionization (MALDI)‐time‐of‐flight mass spectrometry (TOF‐MS), and we obtained a v‐SNARE protein, VAMP8, as a candidate molecule. As shown in Fig. 10, BMMCs lysates were immunoprecipitated with normal or anti‐Rab44 IgG, and the resulting immune complex was subjected to western blotting analysis using an anti‐VAMP8 antibody. The protein that co‐immunoprecipitated with anti‐Rab44 IgG, with a molecular mass of approximately 14 kDa, corresponded to VAMP8, which was not present in the normal rabbit IgG immunoprecipitates (Fig. 10A).

We further performed immunoprecipitation experiments using BMMCs from wild‐type (*Rab44* ^+/+^), *Rab44^+/‐^*, and *Rab44^−/−^* mice. A moderate amount of VAMP8 was observed in *Rab44^+/+^* BMMCs, a smaller amount was detected in *Rab44^+/‐^* BMMCs, and VAMP8 was not detected in *Rab44^−/−^* BMMCs (Fig. 10B). In contrast, when cell lysates were initially immunoprecipitated with an anti‐VAMP8 antibody and subjected to western blotting analysis using an anti‐Rab44 antibody, co‐immunoprecipitates corresponding to both the long and short forms of Rab44 were detected at molecular masses of approximately 160 and 95 kDa, respectively (Fig. 10B). In addition, strong signals were detected in *Rab44* ^+/+^ BMMCs, weak signals were detected in *Rab44* ^+/‐^ BMMCs, and no signal was detected in *Rab4*4^−/−^ BMMCs (Fig. 10B). As shown in Fig. 10C, we confirmed that Rab44 and VAMP8 interaction occurred in both mice and humans. Immunoprecipitation with an anti‐GFP antibody, followed by western blotting with an anti‐VAMP8 antibody, showed that mouse and human Rab44, and their CA mutants, mQ596L and hQ892L, interacted with VAMP8.

We further examined colocalization of Rab44 and VAMP8. hWT was found to only partially colocalize with VAMP8‐positive organelles (Fig. 10D); however, the CA mutant, hQ892L, almost completely colocalized with VAMP‐labeled vesicles (Fig. 10D). Similarly, whereas m‐long colocalized partially with VAMP8‐positive vesicles, the CA mutant mQ795L almost overlapped with VAMP8‐labeled vesicles (Fig. 10D). Quantitative analysis of colocalization of Rab44 and VAMP8 showed that the colocalization scores of hQ892L and mQ795L were significantly higher than those of hWT and m‐long, respectively (Fig. 10E). These results suggest that the active GTP‐bound form of Rab44 preferentially binds to VAMP8.

## Discussion

Native Rab44 localized mainly to lysosomes and the ER and partially to early endosomes in BMMCs. Even in small Rab GTPases, there are only a few proteins that exhibit such widespread localization. For example, Rab10 has been reported to be localized in the ER, Golgi/TGN, endosomes/phagosomes, and GLUT4 vesicles [25]. Therefore, it is unsurprising that Rab44 has a widespread localization in mast cells.

Rab44 is likely to be involved in FcεRI‐mediated release of secretory granules from mast cells. In fact, Rab44‐knockdown BMMCs and BMMCs from *Rab44* ^−/−^ mice reduced FcεRI‐mediated β‐hexosaminidase and histamine secretion *in vitro*. Consistent with these *in vitro* findings, *Rab44* ^−/−^ mice showed impaired anaphylactic responses *in vivo*. However, it is likely that Rab44 is not involved in ionomycin/PMA‐induced exocytosis. Azouz et al. used RBL cells to study the differences between FcεRI‐mediated secretion and secretion in response to costimulation with a Ca^2 +^ ionophore and the phorbolester, TPA [10, 26]. They found that these secretion mechanisms are regulated by different members of the small Rab GTPase family, since, in mast cells, FcεRI‐mediated exocytosis is secretory‐granule specific through the kiss‐and‐run system, while Ca^2+^ ionophore/phorbol ester‐induced exocytosis is a complete secretion process involving immature and mature secretory granules and endosomes [10]. Thus, the present study shows that Rab44 is exclusively responsible for the FcεRI‐dependent release of secretory granules from mast cells.

Several “small” Rab GTPases are known to regulate exocytosis in mast cells. *Rab3D*‐deficient mice have been used to study the effects of Rab3D on mast cell exocytosis, since it is exclusively localized on secretory granules in mast cells [27]. However, *Rab3D*‐deficient mast cells show normal exocytosis from secretory granules, suggesting that other Rab protein(s) control the exocytosis of BMMCs [27]. *Rab27B*‐deficient mice have been shown to have a decreased release of secretory granules from BMMCs [28, 29]. However, even in the absence of *Rab27B*, a considerable amount of secretion from BMMCs remained; therefore, it is assumed that other factor(s) are involved [28, 29]. Rab44 indicated a considerable colocalization with Rab27B in BMMCs in this study, suggesting that they are involved in similar cell events. As such, we propose that Rab44 may control the Rab27B‐independent release of secretory granules from the mast cells. This is based on several lines of evidence: firstly, *Rab44^−/−^* BMMCs exhibited impaired FcεRI‐mediated β‐hexosaminidase secretion, but ionomycin/PMA‐induced exocytosis was not affected. However, *Rab27B*‐knockout BMMCs display decreased β‐hexosaminidase secretion through both FcεRI‐dependent and ionomycin/PMA‐dependent mechanisms [29]. Secondly, based on *Rab44* gene structure, Rab44 contains a Ca^2+^‐binding EF‐hand domain, while Rab27B is a small GTPase without a calcium‐regulating domain. Finally, in BMMCs, Rab44 interacted with VAMP8, whereas Rab27B exclusively interacts with Munc13‐4, which acts as a Ca^2+^ sensor, promoting SNARE complex formation [29]. Considering that the Rab44‐VAMP8 and Rab27B‐Munc13‐4 complexes both have Ca^2+^‐mobilizing functions, it is of interest to determine the details of the functions shared by Rab44 and Rab27B in mast cells.

The release of secretory granules occurs via multiple membrane fusion events that are regulated by the interaction between various v‐SNAREs localized on the granule membrane and t‐SNAREs present on the target membrane. Among the v‐SNAREs, VAMP8 and VAMP7 are important for mast cell degranulation [7]. In mouse mast cells, VAMP8 is crucial for granule fusion during degranulation [30], whereas in human mast cells, both VAMP7 and VAMP8 are required for degranulation [31]. An interaction between Rab44 and the t‐SNARE, VAMP8, was observed in native mouse BMMCs as well as in RBL‐2H3 cells expressing mouse or human Rab44. Therefore, the Rab44‐VAMP8 complex may regulate the release of secretory granules from mouse and human mast cells.

The long and short forms of mouse Rab44 had different localization patterns. The m‐long protein was mainly detected in lysosomes, whereas the m‐short protein was mainly detected in the ER. In addition to the differences between the long and short forms, there were some differences between the mouse long form (m‐long) and the human long form (hWT) of Rab44. The precise mechanisms involved in the differences between m‐long and hWT remain unknown, but differences between m‐long and hWT may be due to the mouse gene lacking the initial section of the coiled‐coil domain and the middle region of the gene (Fig. 2A). These regions missing from mouse Rab44 may be important for the binding of effector(s) that regulate FcεRI‐mediated β‐hexosaminidase secretion and lysosomal formation.

Mutational analyses using CA and DN mutants indicated that the results differed between human and mouse Rab44. The differences are probably due to the different localization patterns between the hWT and m‐long. However, the common feature between human and mouse is that the CA mutants of the human (hQ892L) and mouse (mQ795L) long form both localized at the cell edge. We propose that the inactive long form of Rab44 initially localized on the secretory lysosomes, but after it changes into its active GTP‐bound form, it moves to the edge of the cell. This is consistent with the findings that the DN mutants of human (hT847N) and mouse (mT750N) Rab44 showed increased localization to the lysosomes or early endosomes.

This study also showed that transient Ca^2+^ influx changed Rab44 localization. Both m‐long and hWT proteins partially localized in the lysosomes and then partially translocated to the cytosol and plasma membrane. In contrast, colocalization of m‐short Rab44 with cathepsin D‐positive lysosomes was hardly altered after transient Ca^2+^ influx. Importantly, after ionomycin treatment, the hΔEF mutant was detected as a donut‐like structure, similar to the localization of the hΔEF mutant in nonstimulated cells. Thus, the findings indicating that Rab44 is likely to change its localization may be due to the fact that EF‐hand domain of Rab44 is important for Ca^2+^‐mediated translocation.

## Conclusions

In conclusion, this study demonstrates that mouse Rab44 isoforms increased FcεRI‐mediated β‐hexosaminidase secretion. Both long isoforms localized mainly to lysosomes, whereas the short form localized mainly to the ER. Ionomycin treatment partially alters localization of both long forms, while it hardly alters localization of the short form. Since both Rab44 forms interact with VAMP8, the Rab44‐VAMP8 complex most likely regulates granule exocytosis in mast cells. Thus, Rab44 isoforms similarly promote lysosomal exocytosis but differently localize in mast cells.

## Conflict of interest

The authors declare no conflict of interest.

## Author contributions

TK performed the majority of the experiments; YY, KO, and KO contributed to the construction of expression vectors; MT performed quantitative RT‐PCR; TT supervised the study; TK and TT wrote the manuscript with the contributions of all authors. All authors approved the final manuscript.

## Supporting information


**Table S1.** Primers in this study.
**Fig. S1.** Native Rab44 in RBL‐2H3 cells.
**Fig. S2.** Immunocytochemistry of RBL‐2H3 cells expressing eGFP‐hT847N (A) and eGFP‐mT750N (B).
**Fig. S3.** Original gel image of immunoblot analysis in Figure 1F.
**Fig. S4.** Original gel image of immunoblot analysis in Figure 2C.

## Data Availability

The data that support the findings of this study are available in figures and the supplementary material of this article.

## References

[1] Stenmark H (2009) Rab GTPases as coordinators of vesicle traffic. Nat Rev Mol Cell Biol 10, 513–525. 19603039 10.1038/nrm2728

[2] Hutagalung AH and Novick PJ (2011) Role of Rab GTPases in membrane traffic and cell physiology. Physiol Rev 91, 119–149. 21248164 10.1152/physrev.00059.2009 PMC3710122

[3] Pfeffer SR (2017) Rab GTPases: master regulators that establish the secretory and endocytic pathways. Mol Biol Cell 28, 712–715. 28292916 10.1091/mbc.E16-10-0737 PMC5349778

[4] Srikanth S , Woo JS and Gwack Y (2017) A large Rab GTPase family in a small GTPase world. Small GTPases 8, 43–48. 27221160 10.1080/21541248.2016.1192921 PMC5331893

[5] Yamaguchi Y , Sakai E , Okamoto K , Kajiya H , Okabe K , Naito M , Kadowaki T and Tsukuba T (2018) Rab44, a novel large Rab GTPase, negatively regulates osteoclast differentiation by modulating intracellular calcium levels followed by NFATc1 activation. Cell Mol Life Sci 75, 33–48. 28791425 10.1007/s00018-017-2607-9 PMC11105776

[6] Kadowaki T , Yamaguchi Y , Kido MA , Abe T , Ogawa K , Tokuhisa M , Gao W , Okamoto K , Kiyonari H and Tsukuba T (2020) The large GTPase Rab44 regulates granule exocytosis in mast cells and IgE‐mediated anaphylaxis. Cell Mol Immunol 17, 1287–1289. 32238914 10.1038/s41423-020-0413-z PMC7784977

[7] Wernersson S and Pejler G (2014) Mast cell secretory granules: armed for battle. Nat Rev Immunol 14, 478–494. 24903914 10.1038/nri3690

[8] Elieh Ali Komi D , Wohrl S , Bielory L (2019) Mast cell biology at molecular level: a comprehensive review. Clin Rev Allergy Immunol 58, 342–365. 10.1007/s12016-019-08769-2 31828527

[9] Dahlin JS and Hallgren J (2015) Mast cell progenitors: origin, development and migration to tissues. Mol Immunol 63, 9–17. 24598075 10.1016/j.molimm.2014.01.018

[10] Azouz NP , Hammel I and Sagi‐Eisenberg R (2014) Characterization of mast cell secretory granules and their cell biology. DNA Cell Biol 33, 647–651. 24988214 10.1089/dna.2014.2543 PMC4180300

[11] Luzio JP , Hackmann Y , Dieckmann NM and Griffiths GM (2014) The biogenesis of lysosomes and lysosome‐related organelles. Cold Spring Harbor Perspectives Biology 6, a016840. 10.1101/cshperspect.a016840 PMC4142962 25183830

[12] Woska JR Jr and Gillespie ME (2012) SNARE complex‐mediated degranulation in mast cells. J Cell Mol Med 16, 649–656. 21880114 10.1111/j.1582-4934.2011.01443.x PMC3822836

[13] Puri N and Roche PA (2006) Ternary SNARE complexes are enriched in lipid rafts during mast cell exocytosis. Traffic 7, 1482–1494. 16984405 10.1111/j.1600-0854.2006.00490.x

[14] Lorentz A , Baumann A , Vitte J and Blank U (2012) The SNARE machinery in mast cell secretion. Front Immunol 3, 143. 22679448 10.3389/fimmu.2012.00143 PMC3367400

[15] Goto T , Tsukuba T , Ayasaka N , Yamamoto K and Tanaka T (1992) Immunocytochemical localization of cathepsin D in the rat osteoclast. Histochemistry 97, 13–18. 1618634 10.1007/BF00271276

[16] Nakanishi H , Tsukuba T , Kondou T , Tanaka T and Yamamoto K (1993) Transient forebrain ischemia induces increased expression and specific localization of cathepsins E and D in rat hippocampus and neostriatum. Exp Neurol 121, 215–223. 8339772 10.1006/exnr.1993.1088

[17] Tokuhisa M , Kadowaki T , Ogawa K , Yamaguchi Y , Kido MA , Gao W , Umeda M and Tsukuba T (2020) Expression and localisation of Rab44 in immune‐related cells change during cell differentiation and stimulation. Sci Rep 10, 10728. 32612275 10.1038/s41598-020-67638-7 PMC7329882

[18] Tsuboi T and Fukuda M (2007) Synaptotagmin VII modulates the kinetics of dense‐core vesicle exocytosis in PC12 cells. Genes Cells 12, 511–519. 17397398 10.1111/j.1365-2443.2007.01070.x

[19] Narahara S , Sakai E , Kadowaki T , Yamaguchi Y , Narahara H , Okamoto K , Asahina I and Tsukuba T (2019) KBTBD11, a novel BTB‐Kelch protein, is a negative regulator of osteoclastogenesis through controlling Cullin3‐mediated ubiquitination of NFATc1. Sci Rep 9, 3523. 30837587 10.1038/s41598-019-40240-2 PMC6401029

[20] Kadowaki T , Yukitake H , Naito M , Sato K , Kikuchi Y , Kondo Y , Shoji M and Nakayama K (2016) A two‐component system regulates gene expression of the type IX secretion component proteins via an ECF sigma factor. Sci Rep 6, 23288. 26996145 10.1038/srep23288 PMC4800418

[21] Takii R , Kadowaki T , Tsukuba T and Yamamoto K (2018) Inhibition of gingipains prevents Porphyromonas gingivalis‐induced preterm birth and fetal death in pregnant mice. Eur J Pharmacol 824, 48–56. 29409911 10.1016/j.ejphar.2018.01.028

[22] Blank U , Cyprien B , Martin‐Verdeaux S , Paumet F , Pombo I , Rivera J , Roa M and Varin‐Blank N (2002) SNAREs and associated regulators in the control of exocytosis in the RBL‐2H3 mast cell line. Mol Immunol 38, 1341–1345. 12217405 10.1016/s0161-5890(02)00085-8

[23] Tadokoro S , Nakanishi M and Hirashima N (2010) Complexin II regulates degranulation in RBL‐2H3 cells by interacting with SNARE complex containing syntaxin‐3. Cell Immunol 261, 51–56. 19932892 10.1016/j.cellimm.2009.10.011

[24] Cao J , Ma P , Xue W , Ding Y , Zhang Y and Zhang T (2018) RBL‐2H3 cell model based on VAMP‐8‐EGFP protein for rapid detection of different allergens. Acta Biochim Biophys Sin 50, 1297–1300. 30371727 10.1093/abbs/gmy122

[25] Chua CEL and Tang BL (2018) Rab 10‐a traffic controller in multiple cellular pathways and locations. J Cell Physiol 233, 6483–6494. 29377137 10.1002/jcp.26503 PMC13482492

[26] Azouz NP , Matsui T , Fukuda M and Sagi‐Eisenberg R (2012) Decoding the regulation of mast cell exocytosis by networks of Rab GTPases. J Immunol 189, 2169–2180. 22826321 10.4049/jimmunol.1200542

[27] Riedel D , Antonin W , Fernandez‐Chacon R , Alvarez de Toledo G , Jo T , Geppert M , Valentijn JA , Valentijn K , Jamieson JD , Sudhof TC *et al*. (2002) Rab3D is not required for exocrine exocytosis but for maintenance of normally sized secretory granules. Mol Cell Biol 22, 6487–6497. 12192047 10.1128/MCB.22.18.6487-6497.2002 PMC135623

[28] Mizuno K , Tolmachova T , Ushakov DS , Romao M , Abrink M , Ferenczi MA , Raposo G and Seabra MC (2007) Rab27b regulates mast cell granule dynamics and secretion. Traffic 8, 883–892. 17587407 10.1111/j.1600-0854.2007.00571.x PMC2063611

[29] Singh RK , Mizuno K , Wasmeier C , Wavre‐Shapton ST , Recchi C , Catz SD , Futter C , Tolmachova T , Hume AN and Seabra MC (2013) Distinct and opposing roles for Rab27a/Mlph/MyoVa and Rab27b/Munc13‐4 in mast cell secretion. FEBS J 280, 892–903. 23281710 10.1111/febs.12081

[30] Tiwari N , Wang CC , Brochetta C , Ke G , Vita F , Qi Z , Rivera J , Soranzo MR , Zabucchi G , Hong W *et al*. (2008) VAMP‐8 segregates mast cell‐preformed mediator exocytosis from cytokine trafficking pathways. Blood 111, 3665–3674. 18203950 10.1182/blood-2007-07-103309

[31] Sander LE , Frank SP , Bolat S , Blank U , Galli T , Bigalke H , Bischoff SC and Lorentz A (2008) Vesicle associated membrane protein (VAMP)‐7 and VAMP‐8, but not VAMP‐2 or VAMP‐3, are required for activation‐induced degranulation of mature human mast cells. Eur J Immunol 38, 855–863. 18253931 10.1002/eji.200737634

