# Supplementary material for: Rab44 isoforms similarly promote lysosomal exocytosis, but exhibit differential localization in mast cells
Source: FEBS Open Bio. 2021 Mar 19;11(4):1165–85. doi: 10.1002/2211-5463.13133 (PMC8016136; doi:10.1002/2211-5463.13133)
Supplement: Supplementary file 1 — Table S1. Primers in this study. Fig. S1. Native Rab44 in RBL‐2H3 cells. Fig. S2. Immunocytochemistry of RBL‐2H3 cells expressing eGFP‐hT847N (A) and eGFP‐mT750N (B). Fig. S3. Original gel image of immunoblot analysis in Figure 1F. Fig. S4. Original gel image of immunoblot analysis in Figure 2C. [file FEB4-11-1165-s001.pdf]

Rab44 isoforms similarly promote lysosomal exocytosis, but exhibit differential localization in mast cells

Tomoko Kadowaki, Yu Yamaguchi, Kohei Ogawa, Mitsuko Tokuhisa, Kuniaki Okamoto and Takayuki Tsukuba

Supplementary Table 1. Primers in this study

Sequence Name	Sequence
**Construction of expression vector of wild type Rab44**	
**pMSCV-EGFP-F**	GAATTAGATCTCTCGAGATGGTGAGCAAGGGCGAGGA
**EGFP-hRab44-F**	GGACGAGCTGTACAAGGAGACTGGACAGAGA
**hRab44-EGFP-R**	TCTCTGTCCAGTCTCCTTGTACAGCTCGTC
**hRab44-pMSCV-R**	AATTCGTTAACCTCGAGTCAGGAGCAACAGCCG
**EGFP-mRab44Long-F**	GACGAGCTGTACAAGGAGAAAGGAAAGGGAGTGT
**mRab44Long-EGFP-R**	ACACTCCCTTTCCTTTCTCCTTGTACAGCTCGTC
**pMSCV-mRab44Long-R**	TTCGTTAACCTCGAGTCAGTGGCAGCAGCCAGCTC
**pET28a-mRab44Short-F**	CATATGATGGCCCTCAGTGCTCAC
**pET28a-mRab44Short-R**	CTCGAGGTGGCAGCAGCCAGCTCTC
**Mutagenesis**	
**hRab44_Q892L-F**	CAGCTGGCCTTGAGAGGTACCACAGTATG
**hRab44_Q892L-R**	TACCTCTCAAGGCCAGCTGTGTCCCAGAG
**hRab44_T847N-F2**	GGCAAAAACTCCTTCCTGCACCTGCTG
**hRab44_T847N-R2**	GAAGGAGTTTTTGCCCACGTTGGAGTC
**hRab44-dEF3-F**	CTCTCAGAGGAAGCCACTGCCCTCT
**hRab44-dEF3-R**	GGCTTCCTCTGAGAGGACCAAGACTC
**hRab44-dcoil-F2**	CTTCATGCAGCTACTGAGCAACTTT
**hRab44-dcoil-R2**	GTAGCTGCATGAAGGCAAGGAACGC
**hRab44_836-1021-F**	TGTACAAGCCCCAGGCCAACCCTGAT
**hRab44_836-1021-R**	GCCTGGGGCTTGTACAGCTCGTCCAT
**mRab44Q795L-F2**	GCTGGACTTGAGAGGTACCACAGCCTC
**mRab44Q795L-R2**	CCTCTCAAGTCCAGCTGTGTCCCACAG
**mRab44T750N-F**	GGCAAGAATTCATTCCTACACCTGCTA
**mRab44T750N-R**	GAATGAATTCTTGCCCACATTGGAGT
**Quantitative RT-PCR**	
**mRab44-qF**	AGAGACCACACACACTCTC
**mRab44-qR**	CTCCTGTAAGTCTGTTCTTG
**mGAPDH-qF**	AACGACCCCTTCATTGACCTC
**mGAPDH-qR**	ACTGTGCCGTTGAATTTGCC
**rRab44-qF**	TGGCCAGGCTAGAGAGTCAT
**rRab44-qR**	AGACGTTTGGTCAGGAGCAG
**rGAPDH-qF**	GATCGTGGAAGGGCTAATGA
**rGAPDH-qR**	GAGCTCTGGGATGACTTTGC

Rab44 isoforms similarly promote lysosomal exocytosis, but exhibit differential localization in mast cells

Tomoko Kadowaki, Yu Yamaguchi, Kohei Ogawa, Mitsuko Tokuhisa, Kuniaki Okamoto and Takayuki Tsukuba

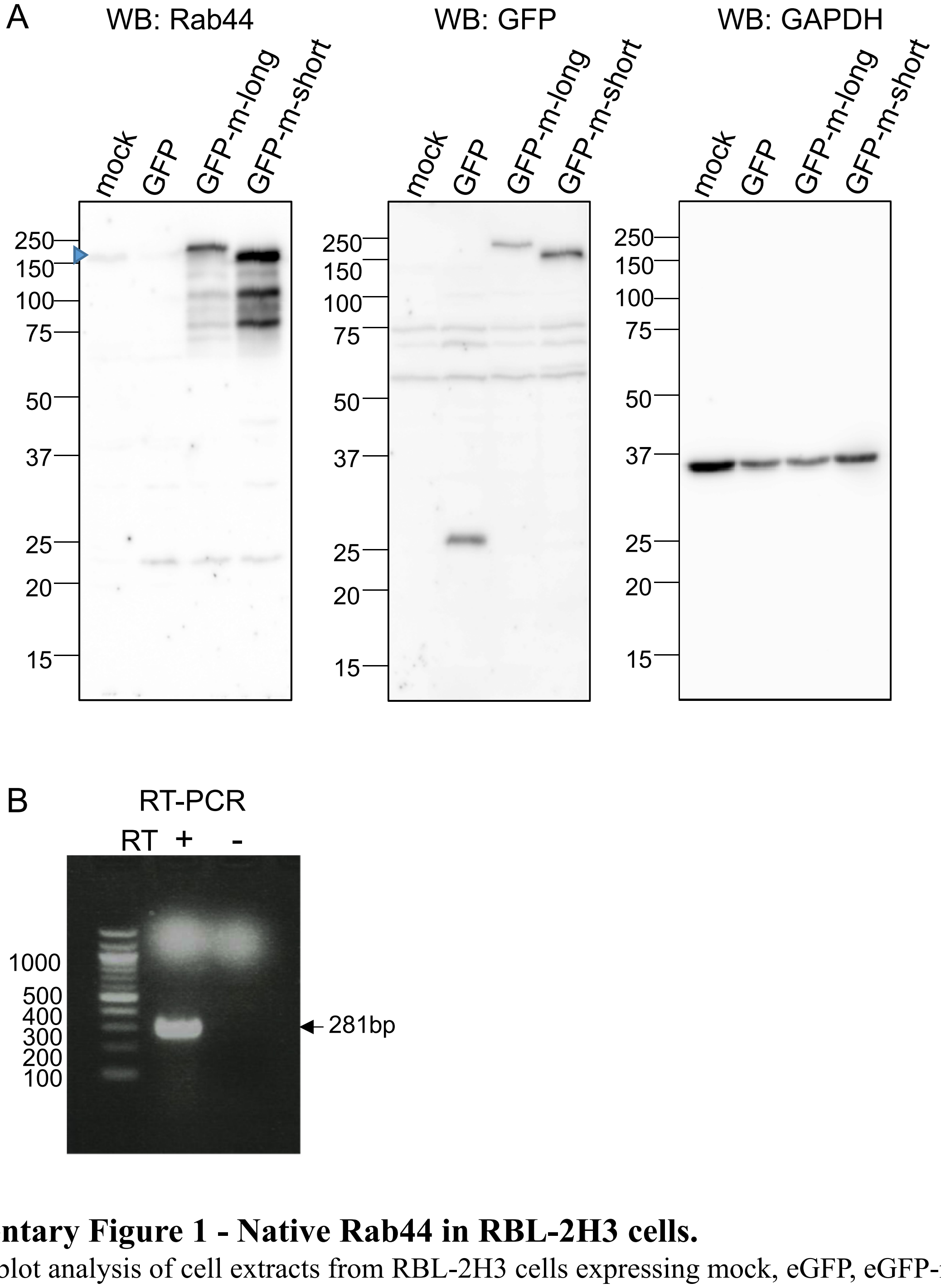

Supplementary Figure 1 - Native Rab44 in RBL-2H3 cells.

A. Western blot analysis of cell extracts from RBL-2H3 cells expressing mock, eGFP, eGFP-m-long, and eGFP-m-short, using anti-Rab44 antibody (A) and anti-GFP antibody (B). Equal protein amount (20ug each) were appled per lane. **B**. RT-PCR of rat Rab44 in RBL-2H3. Primers used were indicated in supplementary Table 1.

Rab44 isoforms similarly promote lysosomal exocytosis, but exhibit differential localization in mast cells

Tomoko Kadowaki, Yu Yamaguchi, Kohei Ogawa, Mitsuko Tokuhisa, Kuniaki Okamoto and Takayuki Tsukuba

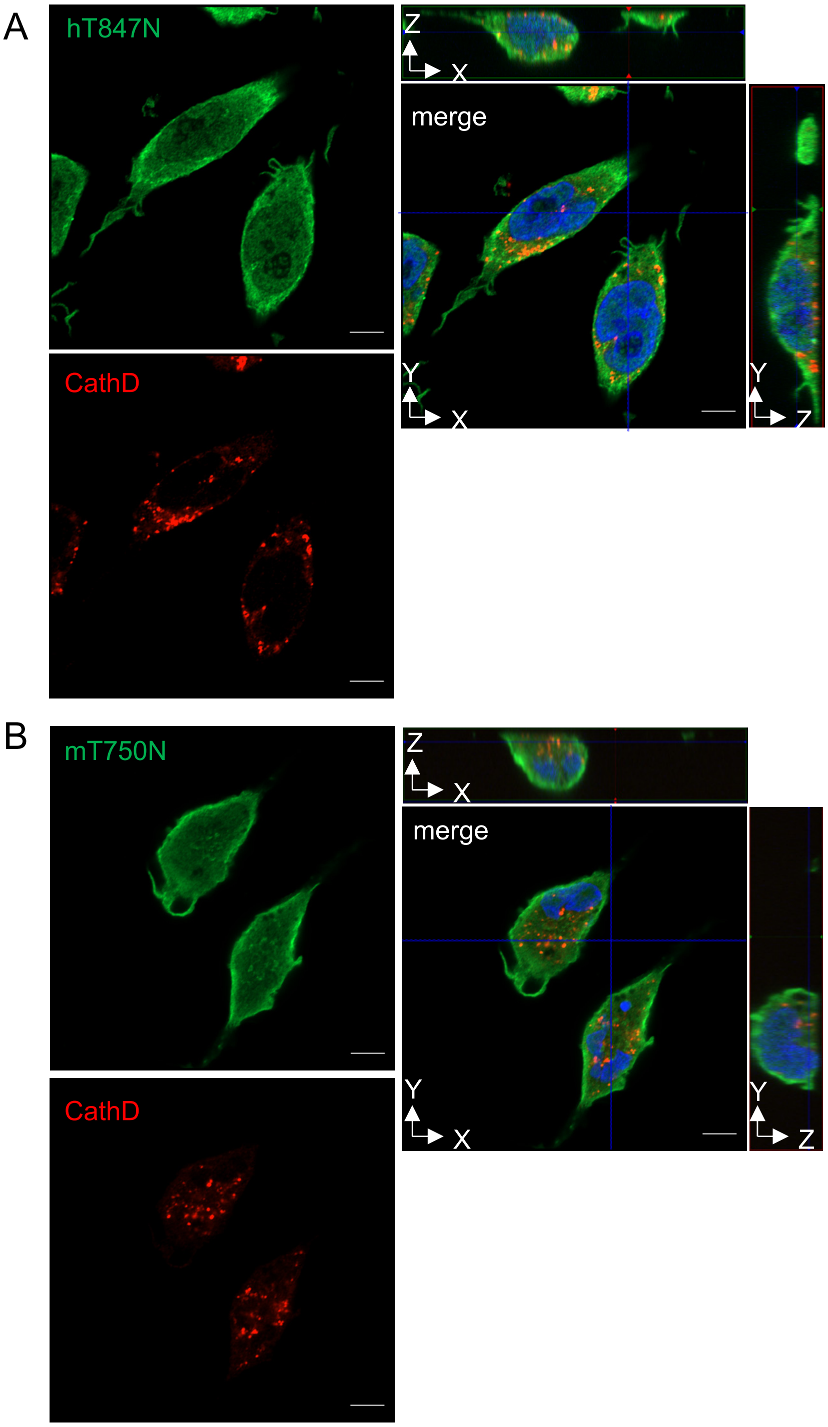

Supplementary Figure 2 - Immunocytochemistry of RBL-2H3 cells expressing eGFP-hT847N (A) and eGFP-mT750N (B).

Cells were immunofluorescently stained with rabbit anti-cathepsin D IgG and mouse anti-GFP IgG, followed by Alexa Fluor 555-conjugated goat anti-rabbit IgG and Alexa Fluor 488-conjugated goat anti-mouse IgG. Bar: 5 µm.

Rab44 isoforms similarly promote lysosomal exocytosis, but exhibit differential localization in mast cells

Tomoko Kadowaki, Yu Yamaguchi, Kohei Ogawa, Mitsuko Tokuhisa, Kuniaki Okamoto and Takayuki Tsukuba

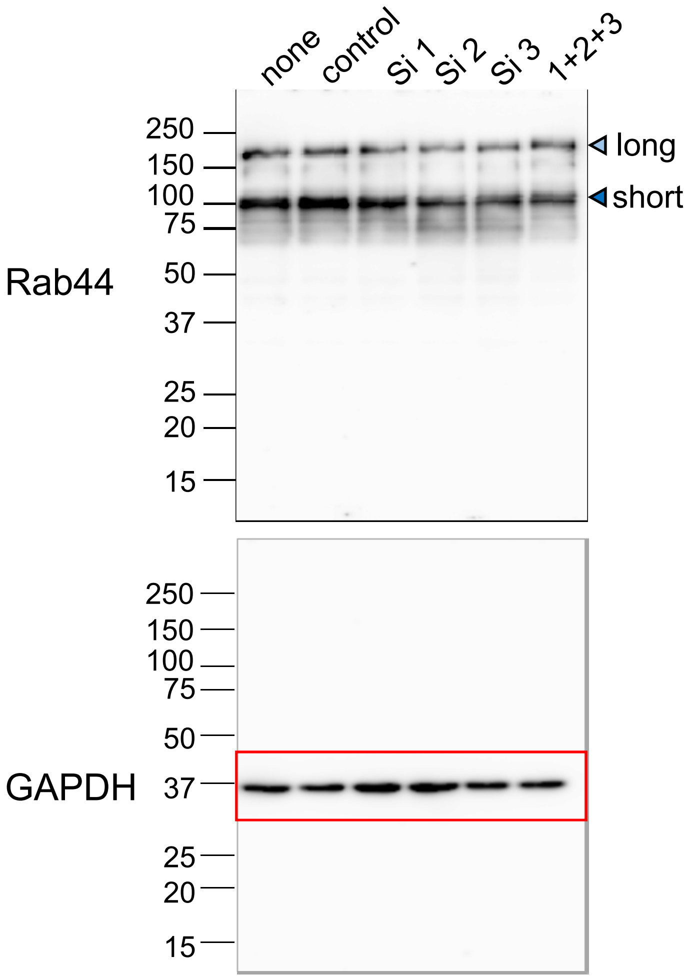

Supplementary Figure 3 – Original gel image of immunoblot analysis in Figure 1F.

Rab44 isoforms similarly promote lysosomal exocytosis, but exhibit differential localization in mast cells

Tomoko Kadowaki, Yu Yamaguchi, Kohei Ogawa, Mitsuko Tokuhisa, Kuniaki Okamoto and Takayuki Tsukuba

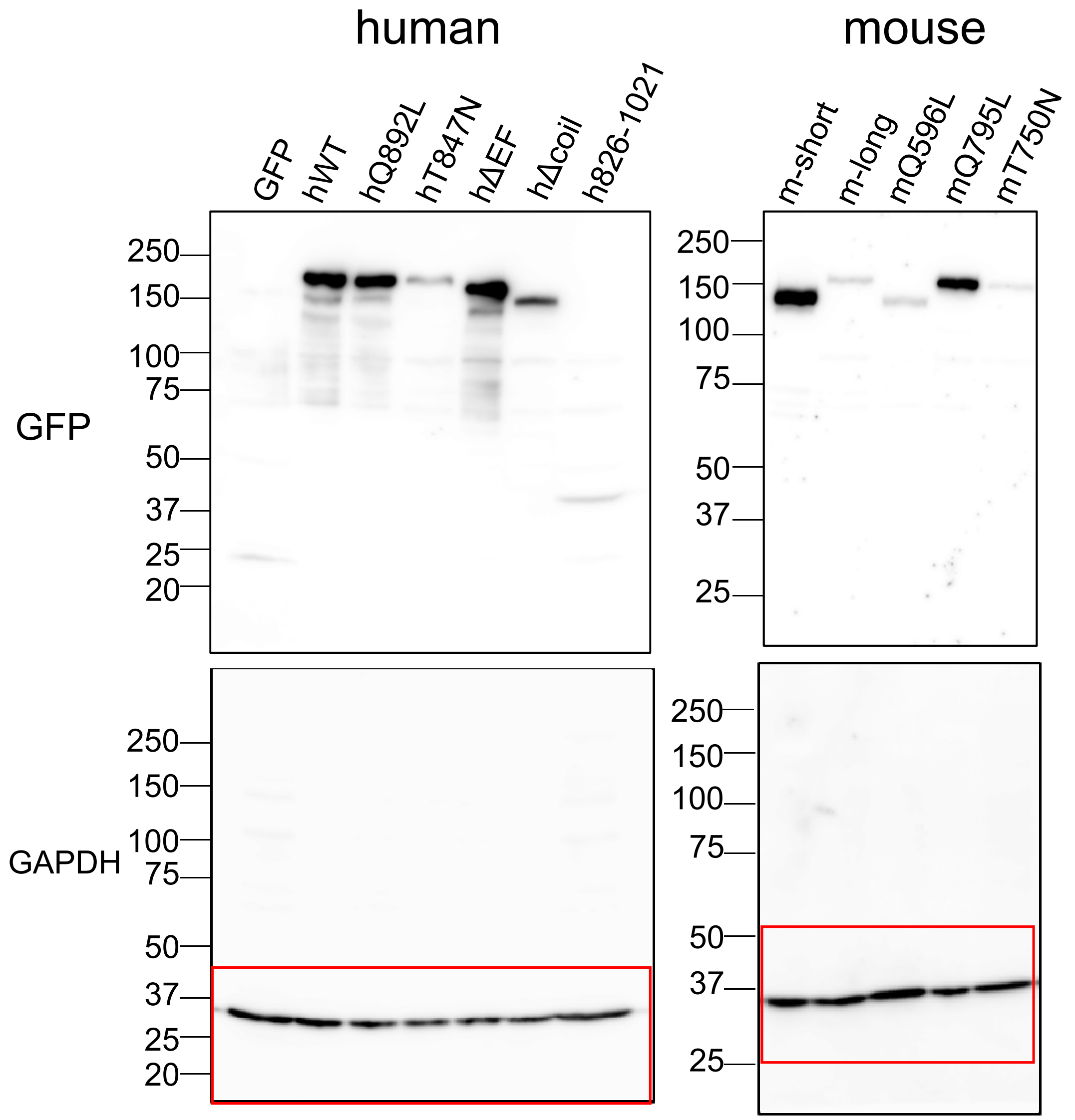

Supplementary Figure 4 - Original gel image of immunoblot analysis in Figure 2C.
